# Unfolding the mysteries of heterogeneity from a high-resolution perspective: integration analysis of single-cell multi-omics and spatial omics revealed functionally heterogeneous cancer cells in ccRCC

**DOI:** 10.18632/aging.205974

**Published:** 2024-06-26

**Authors:** Jie Zheng, Wenhao Lu, Chengbang Wang, Shaohua Chen, Qingyun Zhang, Cheng Su

**Affiliations:** 1 Department of Urology, The First Affiliated Hospital of Guangxi Medical University, Nanning, Guangxi, China; 2 Department of Pediatric Surgery, The First Affiliated Hospital of Guangxi Medical University, Nanning, Guangxi, China; 3 Department of Urology, Guangxi Medical University Cancer Hospital, Nanning, Guangxi, China; 4 Center for Genomic and Personalized Medicine, Guangxi Key Laboratory for Genomic and Personalized Medicine, Guangxi Collaborative Innovation Center for Genomic and Personalized Medicine, Guangxi Medical University, Nanning, Guangxi, China

**Keywords:** clear cell renal cell carcinoma, scRNA, scATAC, spatial transcriptomics, tumor heterogeneous

## Abstract

The genomic landscape of clear cell renal cell carcinoma (ccRCC) has a considerable intra-tumor heterogeneity, which is a significant obstacle in the field of precision oncology and plays a pivotal role in metastasis, recurrence, and therapeutic resistance of cancer. The mechanisms of intra-tumor heterogeneity in ccRCC have yet to be fully established. We integrated single-cell RNA sequencing (scRNA-seq) and transposase-accessible chromatin sequencing (scATAC-seq) data from a single-cell multi-omics perspective. Based on consensus non-negative matrix factorization (cNMF) algorithm, functionally heterogeneous cancer cells were classified into metabolism, inflammatory, and EMT meta programs, with spatial transcriptomics sequencing (stRNA-seq) providing spatial information of such disparate meta programs of cancer cells. The bulk RNA sequencing (RNA-seq) data revealed high clinical prognostic values of functionally heterogeneous cancer cells of three meta programs, with transcription factor regulatory network and motif activities revealing the key transcription factors that regulate functionally heterogeneous ccRCC cells. The interactions between varying meta programs and other cell subpopulations in the microenvironment were investigated. Finally, we assessed the sensitivity of cancer cells of disparate meta programs to different anti-cancer agents. Our findings inform on the intra-tumor heterogeneity of ccRCC and its regulatory networks and offers new perspectives to facilitate the designs of rational therapeutic strategies.

## INTRODUCTION

Clear cell renal cell carcinoma (ccRCC), whose global prevalence and mortality rates have shown significant annual increases, is the most frequent and aggressive histological kidney cancer subtype [1, 2]. Early-stage ccRCC cases are mostly curable with radical resection, however, more than one third of patients develop postoperative disease recurrence with localized or distant metastasis. The insidious clinical manifestations and invasive nature of such disease largely contributes to metastases at initial diagnosis [3]. Despite the advances in pharmacological management of metastatic ccRCC, the survival outcomes for such patients are poor, with 5-year overall survival (OS) outcomes of 10% [4, 5]. Thus, there is a need to investigate the mechanisms involved in regulating tumor invasiveness, which might aid in identification of novel avenues for dissemination of cancer cells in microenvironments.

Intra-tumor heterogeneity, an essential feature in cancer biology and clinical oncology, directly contributes to cancer metastasis, drug resistance, and recurrence [6–8]. Intrinsic or acquired resistance to anti-cancer drugs is a major clinical challenge [4, 9]. In the personalized precision medicine era, ccRCC cases in large-scale cohort studies can be classified into divergent cancer subtypes based on molecular typing. In this scenario, individuals vary in terms of genetic background and tumorigenic phenotypes that contribute to cancer relapse as well as in capacities of cancer cells to respond to treatment modalities [10, 11]. Such precise classification is of significance in providing a conceptual scaffold of revealing the intra-tumor heterogeneity among individual ccRCC patients [12]. Previous studies in this field were focused on bulk RNA-sequencing (RNA-seq), resolution of which is insufficient to investigate the genetic heterogeneity at the single-cell level, masking fateful alterations of transcriptomic spectrum in the most susceptible cell subsets of the tumor microenvironment (TME). Advances in single-cell omics have facilitated the mapping of cellular heterogeneities in an unbiased fashion, thereby independently disclosing cellular identities and functions based on priori defined labeling strategies. The aforementioned molecular classifications based on RNA-seq have been eclipsed by single-cell technologies [13–15]. Functional heterogeneities among cancer cells are not as clearly defined as cellular identities, which involves alterations of the expression spectrum of various genes in disparate functional modules [16, 17]. Molecular classifications of cancer cells are frequently affected by sampling and batch effects [18], and the single-sample based non-negative matrix factorization (NMF) algorithm presents promising performance in disentangling the intricate cellular states in heterogeneous cancer cell subpopulations [17, 19, 20]. Spatial localization of cancer cells will reveal their functional heterogeneities [21, 22]. Given the loss of spatial location information of single cells during tissue dissociation, spatial omics provide an opportunity to overcome this challenge.

We investigated the transcriptomic and epigenomic features of functionally heterogeneous ccRCC cells in the TME by integrating single-cell RNA sequencing (scRNA-seq) and transposase-accessible chromatin sequencing (scATAC-seq) data. Spatial transcriptomics sequencing (stRNA-seq) data was used to obtain spatial information of functionally heterogeneous cancer cells. Our study provides novel approaches for integrating single-cell multi-omics and spatial omics to elucidate on the basic mechanisms of intra-tumor heterogeneity and its associated regulatory networks. Our findings inform on identification of novel therapeutic targets, and crafting of a framework for making personalized treatment decisions for ccRCC patients.

## RESULTS

### Integration analysis of single-cell transcriptome and epigenome profiles of ccRCC

**Figure 1:**
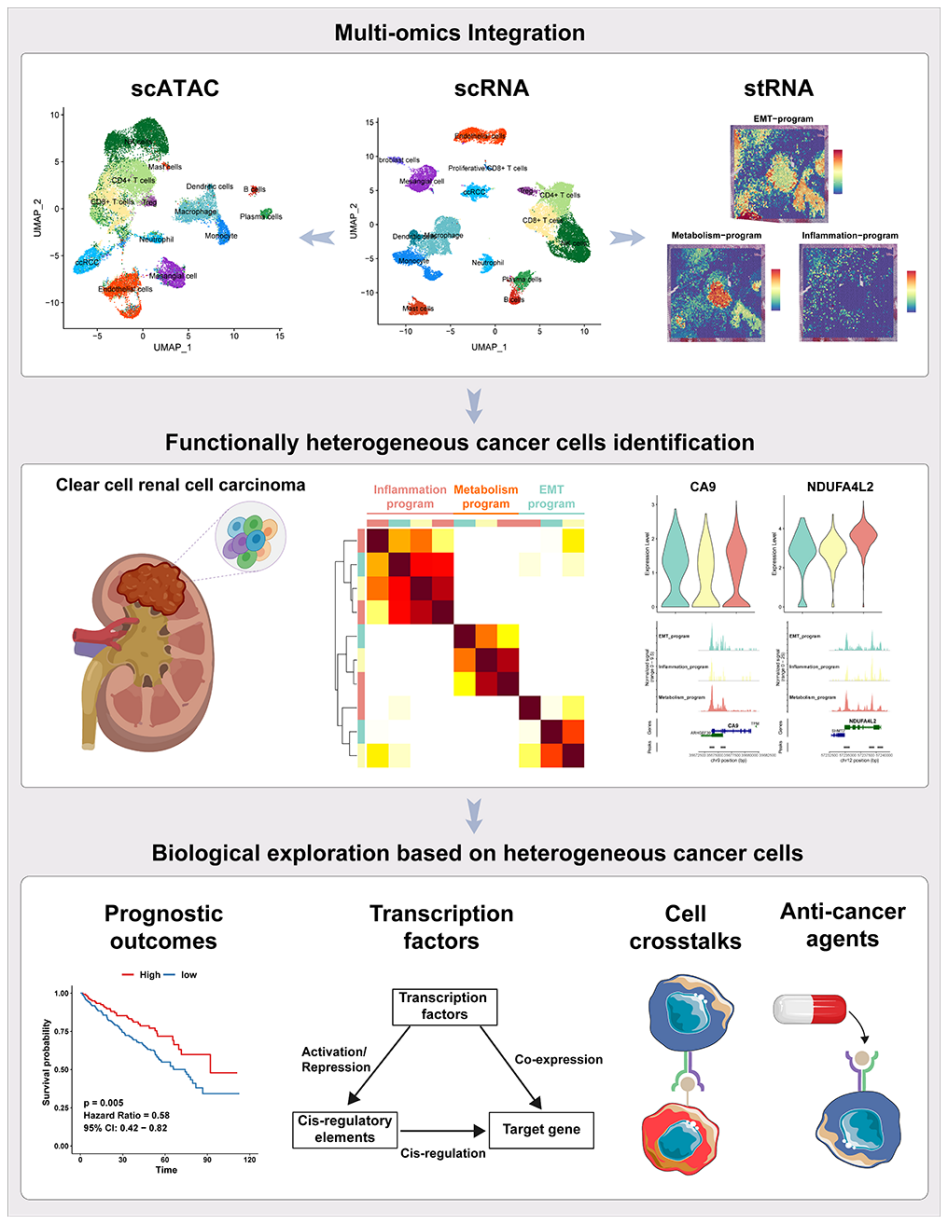
**The workflow of the presented study.** In this study, we approached the functional heterogeneity of cancer cells from a single-cell multi-omics perspective, classifying them into metabolism, inflammatory, and EMT meta programs. Spatial transcriptome sequencing provided spatial information about these distinct meta programs within cancer cells. Bulk-RNA data revealed the high clinical prognostic value of the functional heterogeneity of cancer cells. Transcription factor regulatory networks and motif activities unveiled key transcription factors regulating the functional heterogeneity of ccRCC cancer cells. Interactions between different meta programs cancer cells and other cellular subpopulations in the tumor microenvironment were demonstrated using cellphoneDB. Finally, we assessed the sensitivity of cancer cells from different meta programs to various anticancer drugs.

**Figure 2:**
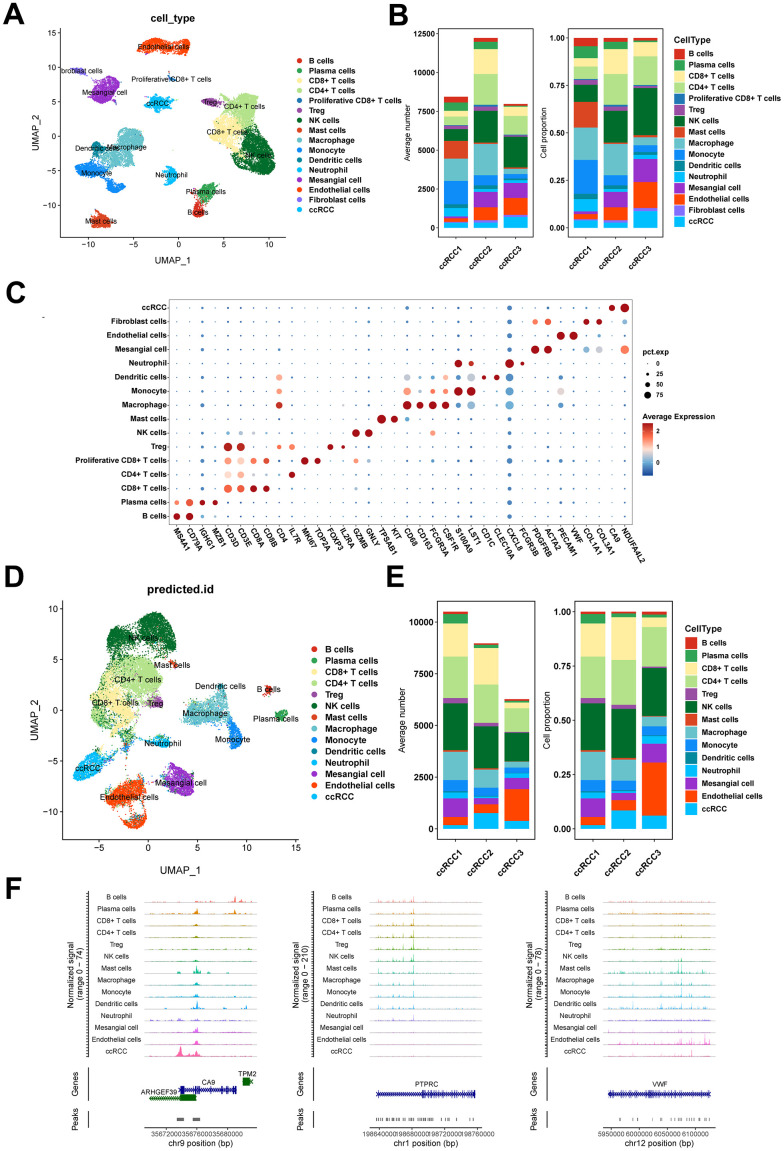
**Single-cell transcriptome and epigenome profiles of ccRCC.** (**A**) UMAP embedding of cells from scRNA-seq data. (**B**) Bar plots showing the number and fraction of each cell type in different samples in scRNA-seq data. (**C**) Dot plot displaying the expression patterns of marker genes for each cell type in scRNA-seq data. (**D**) UMAP embedding of cells from scATAC-seq data. (**E**) Bar plots showing the number and fraction of each cell type in different samples in scATAC-seq data. (**F**) Chromatin accessibility profiles of marker genes for each cell type in scATAC-seq data.

The workflow of the presented study was shown in Figure 1. To dissect the transcriptomic and epigenomic profiles of ccRCC at single-cell resolution, scRNA-seq and scATAC-seq data were downloaded from the NCBI SRA database, with accession number PRJNA768891 (for information of data see Supplementary Table 1). After quality control, a total of 28,639 single-cell transcriptomes were retained for subsequent analyses (Supplementary Figure 1A, 1B). Cell identities were defined using canonical marker genes as previously described [18, 23, 24]. Sixteen cell types were identified in scRNA-seq data (Figure 2A). The average number and relative proportions of each cell type are shown in Figure 2B. Expression levels of marker genes of corresponding cell types are presented in Figure 2C. A total of 25,733 cells were generated from scATAC-seq data after quality control filtering (Supplementary Figure 1C, 1D). Cell identities of scATAC-seq were annotated in a supervised manner based on Seurat’s label-transfer algorithm (Figure 2D and Supplementary Figure 1E). Single-cell epigenome profiles were perfectly assigned to specific cell types (Figure 2D, 2E), which was verified by normalized chromatin accessibility profiles for each cell type and marker genes (Figure 2F). Most of the cell clusters in scATAC-seq were mapped to their corresponding cell subpopulations in scRNA-seq, apart from proliferative CD8^+^ T and fibroblast cells, in tandem with research of the data source [23]. We also defined two distinct cell types, viz., fibroblast cells predominantly expressing COL1A1 and COL1A3 in scRNA-seq, as well as plasma cells highly expressing IGHG1 and MZB1 in scRNA and scATAC-seq. Relative abundances of cell types, particularly endothelial cells and ccRCC cells, exhibited marked differences between samples (Figure 2B, 2E), highlighting inter-tumoral heterogeneity among individual ccRCC patients.

### Single-cell multi-omics and spatial omics revealed functionally heterogeneous ccRCC cells

**Figure 3:**
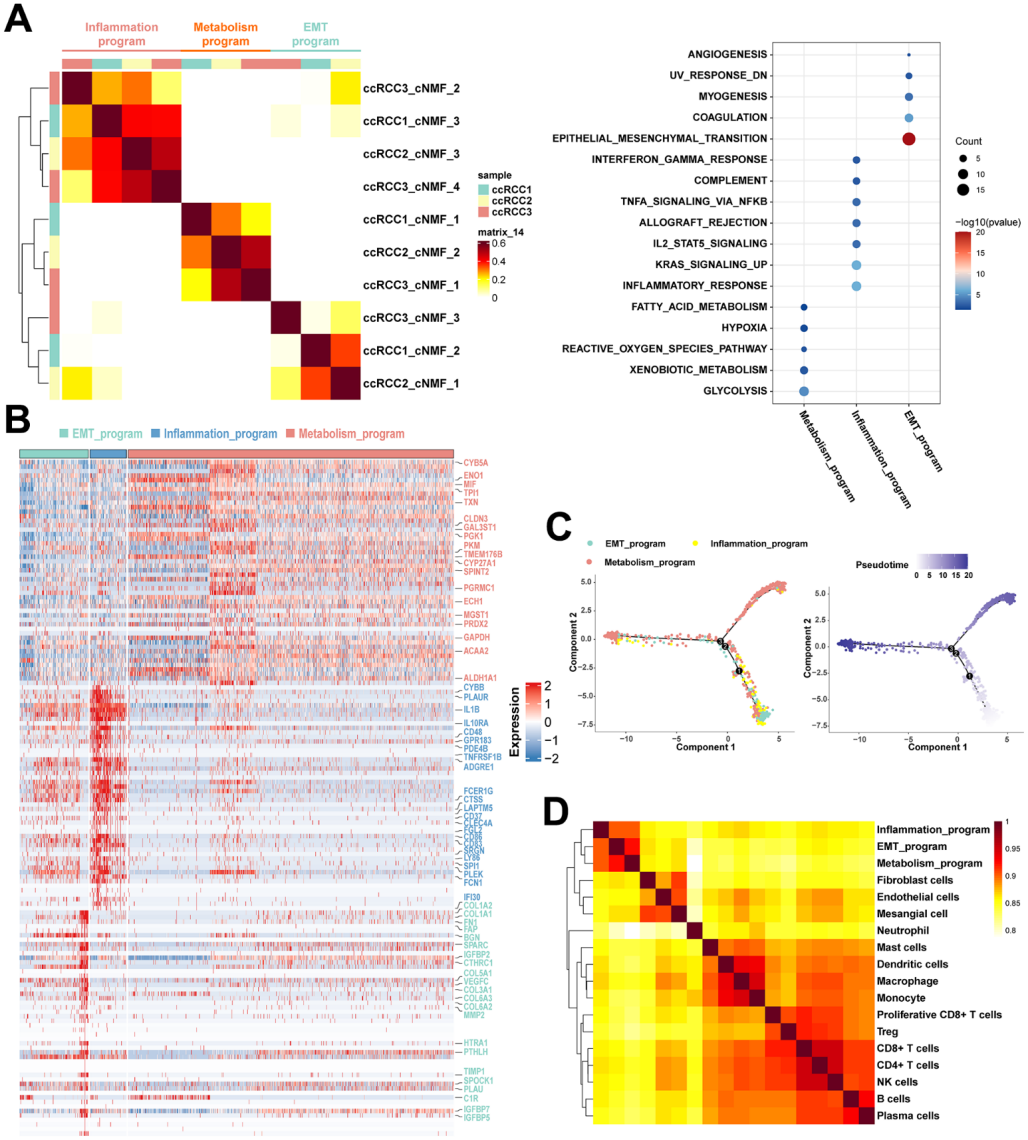
**Functionally heterogenous cancer cells in ccRCC.** (**A**) Heatmap of pairwise correlations of ten intra-tumoral programs derived from three ccRCC samples, with the right plot revealing functional characteristics of different meta programs based on functional enrichment analysis. (**B**) Heatmap showing the specific characteristic gene expression of three meta programs of functionally heterogenous cancer cells. Characteristic genes of three meta programs were marked by the color. (**C**) Pseudotime analysis of three meta programs of functionally heterogenous cancer cells based on Monocle2. The numbers 1, 2, and 3 represent node changes in cell differentiation, where nodes 1 and 2 have fewer branches, while node 3 is the main node of cell differentiation. (**D**) Pairwise correlation plot of cell types identified in scRNA-seq data, illustrating similarities between different cell types in ccRCC.

To resolve the heterogeneity of ccRCC cells in multi-omics, we analyzed the cancer cells in the TME. The NMF algorithm is advantageous in interpreting tumor heterogeneity, marginally influenced by batch effects, thereby obtaining factors with biological interpretability via non-negative matrix factorization. The cNMF algorithm implemented in python [25] showed that cancer cells from the three ccRCC cases could be classified into ten programs (Supplementary Figure 2A, 2B). Based on similarities between programs, three meta programs covered by all ccRCC cases were identified (Figure 3A), which reflected the complicated phenotypes of functionally heterogeneous cancer cells in ccRCC TME. There were significant variations in transcriptomic profiles of such meta programs, all of which were functionally defined as inflammatory meta program, metabolism meta program, and EMT meta program based on pathway enrichment analysis of their specific characteristic gene expression signatures (Figure 3A, 3B and Supplementary Tables 2, 3). The inflammatory meta programs were mainly enriched in inflammatory responses, complement, interferon gamma responses, and IL2 STAT5 signaling pathway. The EMT meta program was significantly enriched in epithelial mesenchymal transition, coagulation, myogenesis, and angiogenesis, while metabolism meta program was highly enriched in hypoxia, xenobiotic metabolism, glycolysis, fatty acid metabolism, and reactive oxygen species pathway. The metabolism meta program accounted for the largest proportion of cancer cells, implying that ccRCC is a hypermetabolic disease, as evidenced by abnormal accumulation of lipid droplets in cancer tissues and cell lines, and its close association with lipid metabolism pathways [24, 26]. Annotation of cancer cell clusters in scRNA data with corresponding clusters in scATAC data revealed that the three meta programs manifested elevated expression and chromatin opening (Supplementary Figure 3A) of cancer-specific markers (*CA9* and *NDUFA4L2*). There was a loss of chromosome 3p and amplification of chromosome 5q in all three meta programs (Supplementary Figure 3B). Pseudotime analysis showed that cancer cells in EMT meta programs were at the beginning of the differentiation trajectory, while cancer cells of the metabolism meta program were predominant at the trajectory terminus, representing the specific physiological states of the functionally heterogeneous cancer cells in the TME. Thus, EMT is a crucial process in cancer developments (Figure 3C).

**Figure 4:**
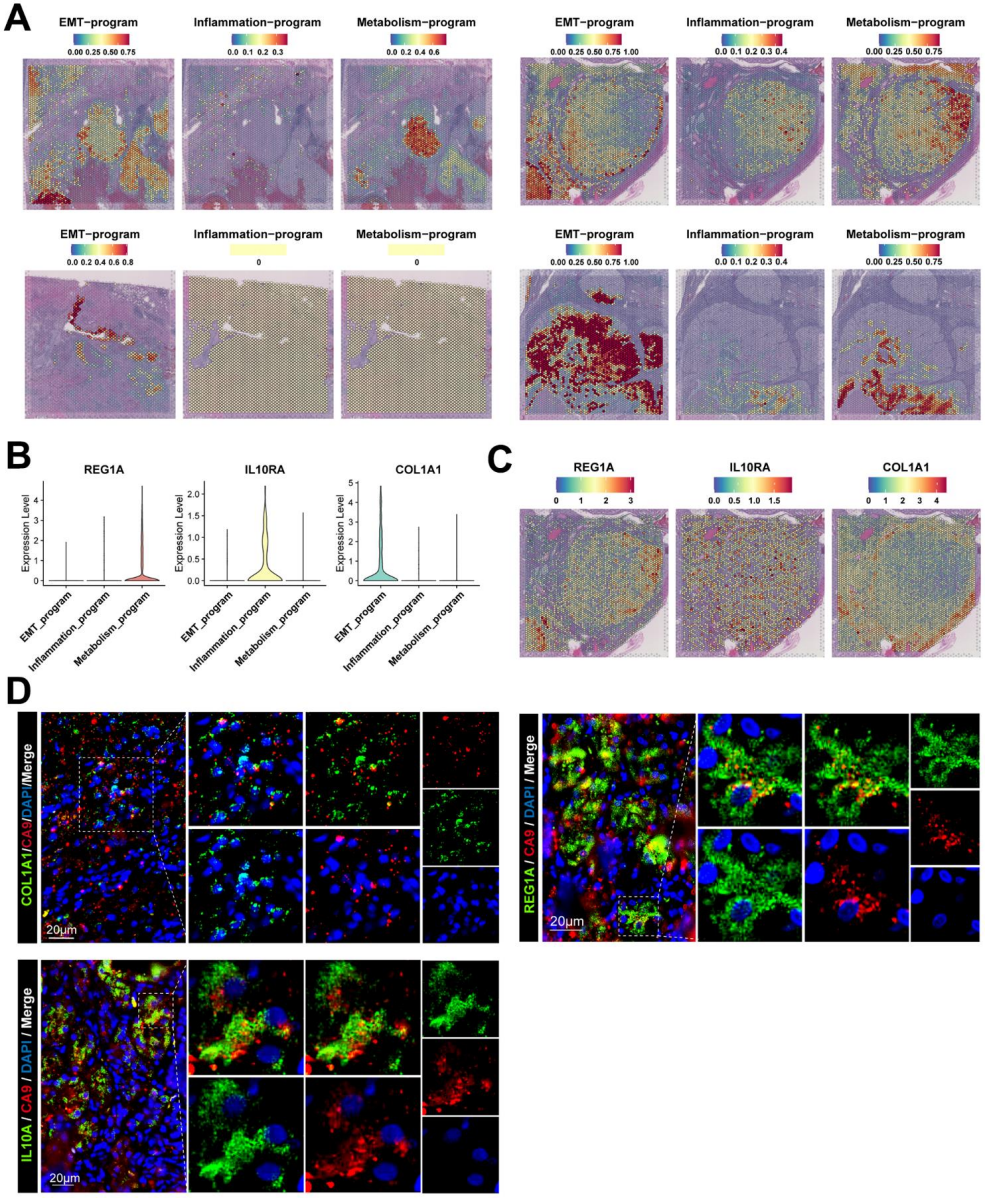
**Spatial location information and signature gene characteristics of functionally heterogeneous cancer cells in ccRCC.** (**A**) Spatial location information of functionally heterogeneous cancer cells revealed by stRNA-seq, illustrating the distribution of cancer cells in tumor lesions. (**B**) Signature gene characteristics of functionally heterogeneous cancer cells in scRNA-seq data, showing the expression levels of signature genes of different meta programs. (**C**) Spatial expression patterns for signature gene characteristics of functionally heterogeneous cancer cells in stRNA-seq data. (**D**) Signature gene characteristics of functionally heterogeneous cancer cells validated by clinical samples of ccRCC using immunofluorescence.

There were high correlations between disparate meta programs. Stromal cells, i.e., fibroblasts, endothelial cells and mesangial cells exhibited a high degree of similarity. Moreover, various immune cells exhibited similarities. These results suggest that even though cancer cells of the EMT meta program and inflammatory meta program were functionally and phenotypically close to stromal cells and immune cells, respectively, they still have significant transcriptomic variations in their nature (Figure 3D). Due to the loss of spatial location information of scRNA-seq during tissue disassociation, we spatially localized the cancer cells of different meta programs using spatial transcriptomics (Figure 4A). Cancer cells of the EMT meta program were uniformly distributed at margins of tumors and perivascular areas, and such geographic location corroborates the functional definition of EMT meta programs. We hypothesized that ccRCC cells can activate the EMT through close contacts with stromal cells in local environments, and specified cancer cells of identical meta program in perivascular areas synergistically contribute to hematogenous dissemination via the vascular system. The metabolism meta program was mainly located in the tumor center, which could be a consequence of hypoxia and xenobiotic metabolism. We parsed the spatial transcriptomics, combing with single-cell multi-omics data, thereby elucidating on key functions assumed by heterogeneous ccRCC cells.

Transcriptional characterization of signature genes in the three meta programs was performed and validated using the scATAC-seq and stRNA-seq data (Figure 4B, 4C and Supplementary Figure 4A–4D), with results disclosing that the metabolism meta program specifically expressed *REG1A*, a molecular marker for ccRCC [27]. The inflammatory meta program exhibited high expression of *IL10RA*, which is closely associated with interferon receptors and mediates immunosuppressive signaling of *IL10*, thereby inhibiting pro-inflammatory cytokine secretion [28]. The EMT meta program characteristically expressed *COL1A1*, which is preferentially expressed in fibroblasts. Transcriptional profiles of the EMT process suggests that such cancer cells may have fibroblast-like phenotypes and an ability to secrete fibronectin [29]. The three characteristic genes are barely expressed in major cell types of normal kidney tissues (Supplementary Figure 4E–4G). We validated the staining of signature genes of such three meta programs using tumor samples collected in clinical settings (Figure 4D). The signature genes were co-localized with the ccRCC-specific marker (CA9) in cancer tissues [30], revealing the existence of such three types of functionally heterogeneous cancer cells in the TME of ccRCC cases.

### Infiltrations of functionally heterogeneous cancer cells were closely associated with prognosis of ccRCC patients

**Figure 5:**
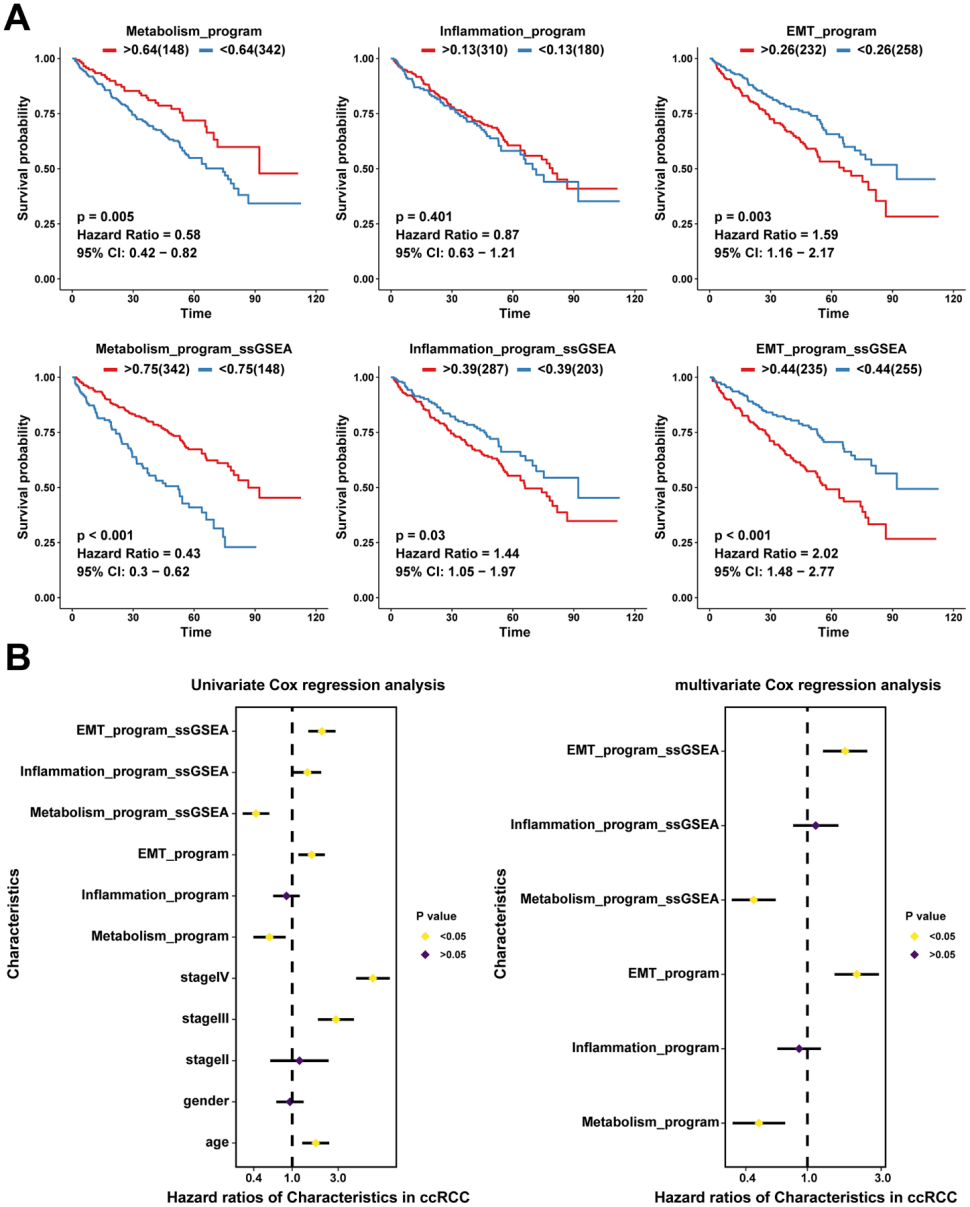
**Clinical values of functionally heterogeneous cancer cells in ccRCC.** (**A**) Kaplan-Meier survival analysis of TCGA ccRCC cohort based on infiltration levels calculated by CIBERSORTx analysis (up) and ssGSEA score calculated by characteristic genes (down) of functionally heterogeneous cancer cells. The grouped metrics are based on the optimal cutoff value, and the numbers in parentheses represent the ccRCC sample size. (**B**) Univariate and multivariate Cox analyses showing the associations between clinical indicators (age, gender, and stage), infiltration levels and ssGSEA scores with patients’ survival outcomes (the prognostic value of functionally heterogeneous cancer cells of the three meta programs).

We assessed the relative abundance of the three meta programs in 490 ccRCC cases from the TCGA database using CIBERSORTx, thereby revealing the associations between the functionally heterogeneous cancer cells and survival outcomes of patients. The metabolism meta program cases were highly abundant in the TCGA cohort, followed by EMT and inflammatory meta programs (Supplementary Figure 5A). Higher infiltration levels of heterogeneous cancer cells of the metabolism meta program were associated with favorable prognostic outcomes, whereas infiltrations of the EMT meta program were associated with poor outcomes (Figure 5A). However, there were no significant correlations between ccRCC cases in the inflammatory meta program and prognostic outcomes of patients. Further, we performed ssGSEA analysis based on characteristic genes (Supplementary Table 3) of each meta program and explored the relationships between ssGSEA scores and prognostic outcomes. There were significant associations between ssGSEA scores of characteristic genes in each meta program and prognosis of ccRCC patients (Figure 5A).

We further performed univariate and multivariate Cox analyses for the effects of cancer cell infiltration abundance, ssGSEA scores of characteristic genes, and clinical indicators (age, gender, and stage) on prognostic outcomes. Univariate Cox analysis revealed identical trends as those obtained from the Kaplan-Meier survival analysis (Figure 5B). After excluding the effects of confounding factors, such as age, gender, and stage, we performed multivariate Cox analysis on ssGSEA scores and infiltration abundance of cancer cells of different meta programs, which showed that infiltration abundance as well as ssGSEA scores of meta metabolism and EMT meta programs were independent prognostic factors; the EMT meta program played an adverse role, while the metabolism program played a protective role in survival outcomes of ccRCC (Figure 5B). The ssGSEA scores were significantly correlated with infiltration abundance of cancer cells in different meta programs, indicating that ssGSEA scores can reflect, to some extent, the infiltration levels of functionally heterogeneous cancer cells (Supplementary Figure 5B).

### Identification of specific transcription factors of functionally heterogeneous cancer cells

**Figure 6:**
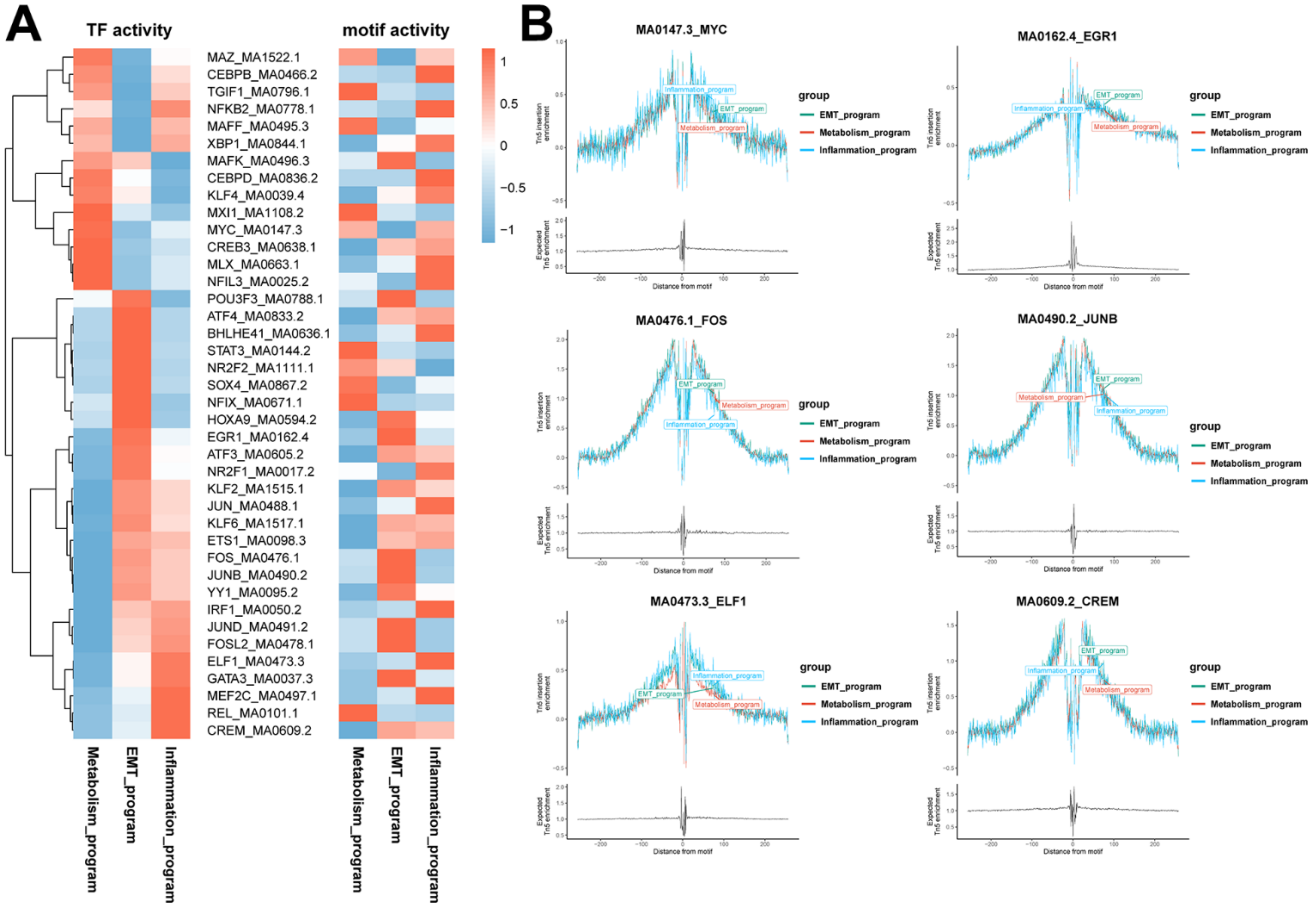
**Identification of specific transcription factors of functionally heterogeneous cancer cells in ccRCC TME.** (**A**) Heatmap of the results of pySCENIC analysis in scRNA-seq data, revealing the putative transcription factors associated with different meta programs. The right heatmap shows motif activities in scATAC-seq data, indicating the activities of transcription factor binding motifs in different cell types. (**B**) Transcription factor footprint analysis of disparate meta programs. The footprint analysis is for computing the normalized Tn5 insertion frequency for each position surrounding AP-1 motif instances. Different colors represent different meta programs.

Program-specific transcription factors were identified by combining scRNA and scATAC data. First, we investigated the transcription factor regulatory network of functionally heterogeneous cancer cells in different meta programs based on scRNA-seq data using pySCENIC. Transcription factor motif activities were inferred using chromVAR packages based on scATAC-seq data, whereby chromatin accessibility of transcription factor binding sites were characterized at the DNA level. Program-specific transcription factors were identified using both pySCENIC and motif activity analysis combined with transcription factor footprint analysis (Figure 6A, 6B). In the metabolism meta program, we identified transcription factor *MYC*, with results of co-expressed downstream target genes of transcription factors showing that it was closely associated with the hypoxia-related signaling pathway. In the EMT meta program, we identified transcription factors *EGR1*, *FOS* and *JUNB*, with downstream target genes being associated with epithelial mesenchymal transition, myogenesis, and the angiogenesis signaling pathway. Moreover, transcription factors *ELF1* and *CREM* were found in the inflammatory meta program, with downstream target genes being predominantly enriched in inflammatory responses, complement, interferon gamma responses, and IL2 STAT5 signaling pathways (Figure 6A, 6B and Supplementary Figure 5C).

### Construction of intercellular communication networks of functionally heterogeneous cancer cells and other cell identities in the TME

**Figure 7:**
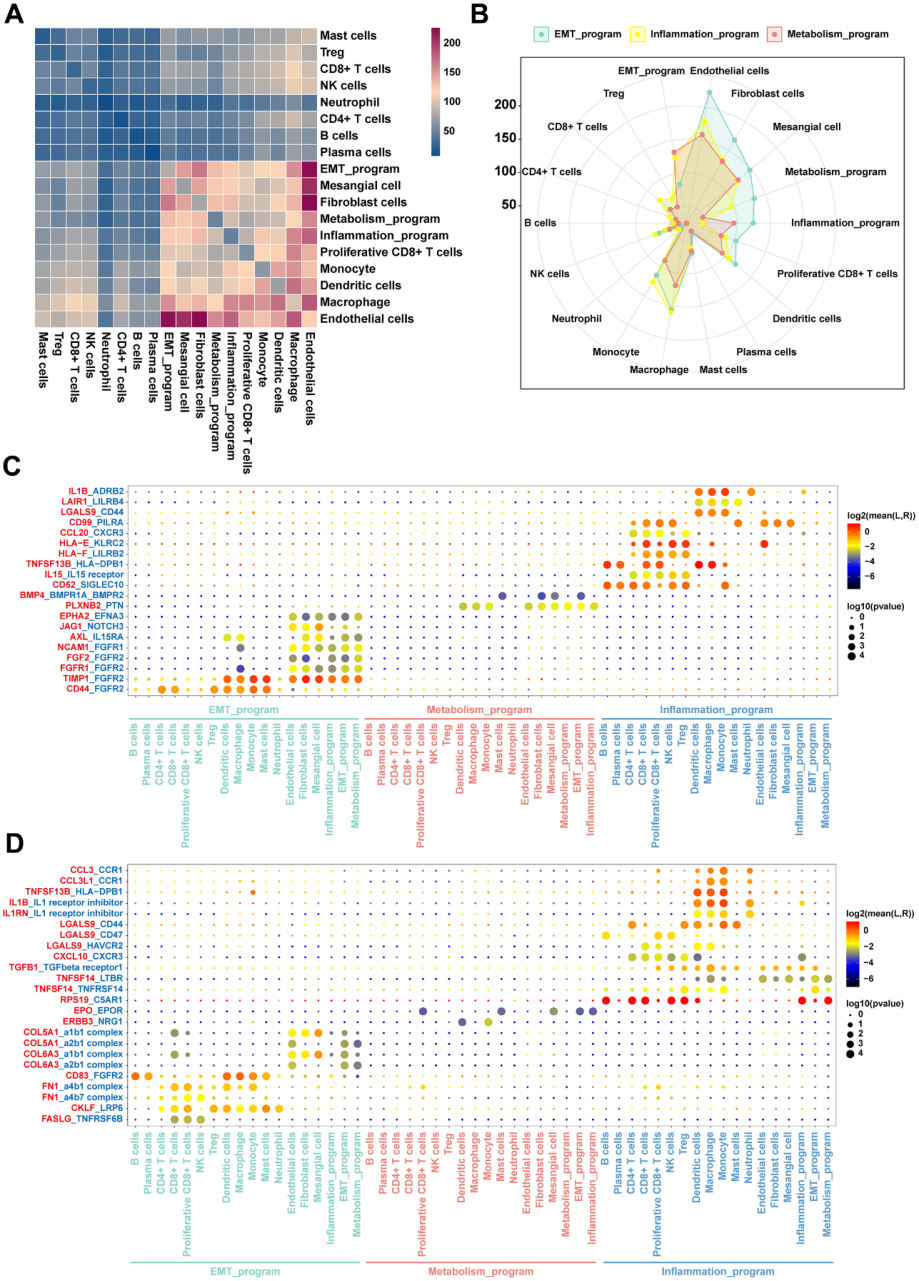
**Intercellular communication networks orchestrated by functionally heterogeneous cancer cells and other cellular identities in ccRCC TME.** (**A**) Heatmap plot of the number of ligand-receptor pairs across disparate cell clusters. (**B**) Radar plot of quantitative comparisons of ligand-receptor pairs across cancer cells in disparate meta programs. (**C**) Dot plots of mean interaction strengths of ligand-receptor pairs between functionally heterogeneous cancer cells, with dot sizes indicating the P-value and colored by the average expression level of the receptor in cancer cells. The plot for expression levels of the receptor in functionally heterogeneous cancer cells. (**D**) The plot showing the expression levels of the ligand in functionally heterogeneous cancer cells.

To establish the crosstalks among various cellular identities in the TME, we investigated ligand-receptor pair expression in different cell clusters using CellphoneDB. We specifically focused on interactions between functionally heterogeneous cancer cells of different meta programs and other cell subpopulations in the microenvironment. Cancer cells of the EMT meta program exhibited abundant ligand receptor pairs with endothelial cells, fibroblasts, proliferative CD8^+^ T cells, dendritic cells and mesangial cells (Figure 7A), and there were more interactions with such cell types when compared with other meta programs (Figure 7B). This shows the close intercellular crosstalk between cancer cells of the EMT meta program with other cell identities in the TME. Both immune and mesangial cells interacted with cancer cells of the EMT program via the FGFR2 receptor of the FGF family (Figure 7C), with evidence indicating the pivotal role of FGFR2 in mediation of EMT and cancer cell angiogenesis [31]. The EMT program exhibited a unique secretory phenotype, characterized by powerful abilities of secreting collagen fibers and enriched communication with mesangial cells (Figure 7D), which were directly associated with cell migration and focal adhesion regulation. The inflammatory meta program presented highly enriched crosstalks with most immune cells, when compared with other meta programs (Figure 7B). Ligand LGALS9 with its receptors (CD44, CD47, and HAVCR2) showed that the inflammatory meta program also interacted with immune cells, implying that it is a cysteine/galactose binding protein that can compromise the functions of NK and T cell to facilitate cancer cell immune escape [32]. Ligands of IL1 receptor inhibitor with receptors of IL1RN and IL1B shared similar roles in immune escape and tumor metastasis (Figure 7D).

Crosstalks and phenotypic descriptions between functionally heterogeneous cancer cells of disparate meta programs and other cell populations were validated using spatial information provided by stRNA-seq. The EMT meta program exhibited stronger abilities in interacting with other cell types in the TME, with their distribution being located at tumor margins and perivascular areas. Interactions of the metabolism meta program were also demonstrated by their locations in the center of the tumor, which was corroborated by attenuations of interactions between cancer cells of the metabolism meta program and other cell identities in the TME.

### Sensitivity assessment of anti-cancer agents to functionally heterogeneous cancer cells

**Figure 8:**
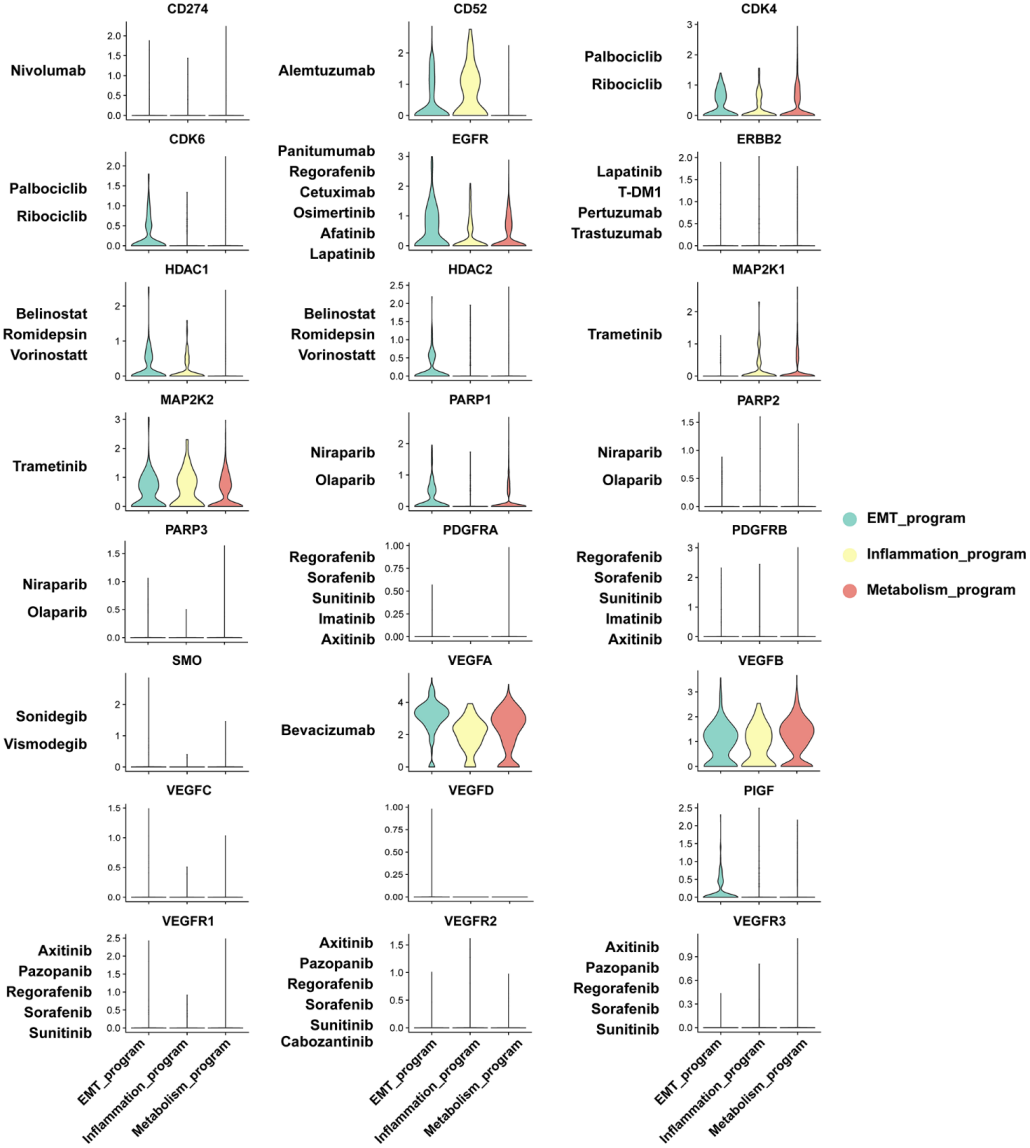
Sensitivity analysis of anti-cancer agents to functionally heterogeneous cancer cells based on expression level of therapeutic target genes.

We investigated the sensitivity of functionally heterogeneous cancer cells in different meta programs to anticancer agents based on therapeutic target genes. We focused on mainstream drugs currently in clinical trials and Food and Drug Administration (FDA)-approved drugs for clinical management of ccRCC. The functionally heterogeneous cancer cells presented varying responses to various anticancer drugs. Targeted drugs for VEGFA have been studied in kidney cancer. Other polypeptide factors that share similar functions and structural homology to VEGFA have been recently identified, spanning placenta growth factor (PIGF), VEGF-B, VEGF-C, VEGF-D and VEGF-E, constituting the VEGF family [33]. VEGFA was highly expressed in different meta programs, indicating that such cell subpopulations were susceptible to Bevacizumab. Expression of VEGFB were also elevated in cancer cells. Antitumor drugs targeting VEGFB have not been reported, however, based on structural similarities between VEGFB and VEGFA, development of targeted drugs against VEGFB has tremendous potential for ccRCC management. Likewise, CDK4, EGFR, and MAP2K2 were highly expressed in the three meta programs, all of which play critical roles in development of anticancer agents (Figure 8).

Physiologically, CD52 is a cell surface glycoprotein that is expressed on mature lymphocytes. Its associated monoclonal antibodies, anti-CD52, including alemtuzumab and analogues, are intended for treatment of multiple sclerosis and B cell chronic lymphocytic leukemia [34], with its specific expression patterns being observed in the inflammatory meta program. The therapeutic target genes, such as CDK6, HDAC2 and PIGF, exhibited enhanced expression levels in the EMT meta program, confirming that drugs targeting these genes may have significant therapeutic responses in such cancer cell subpopulations. Most of the therapeutic target genes were exclusively expressed on functionally heterogeneous cancer cells, with the exception of ERBB2 expression in collecting duct principal cells of normal renal epithelium (Supplementary Figure 6). These results elucidate on the mechanisms underlying the biological significance of functionally heterogeneous cancer cells in the TME, thereby providing a framework for studying cell-cell interactions and drug effects in ccRCC microecosystems.

## DISCUSSION

Intra-tumor heterogeneity is pertinent to metastasis, recurrence, and therapeutic resistance of cancer, which are closely associated with variations in survival rates within cancer patients [6–8]. In this study, we assessed the transcriptomic and epigenetic landscape of ccRCC using scRNA and scATAC data, functionally characterizing heterogeneous cancer cells on multiple dimensions, combined with stRNA-seq to project the spatial orientation of divergent cancer cells and assess their biological variations in the TME. Currently, due to complex expression patterns of functional module genes, elucidation of functional states of cancer cells is in the infancy [16, 17]. The cell subpopulations identified by conventional dimensionality reduction analysis (e.g., Seurat) in single-cell analysis are vulnerable to sampling bias or integration [35], and the established transcriptomic profiles could not reflect the biological characteristics of cancer cells, masking the essential aberrations and slightly observable transcriptomic spectrum in susceptible cell subpopulations [18]. We found that cancer cells in the ccRCC TME can be classified into three meta programs using the cNMF algorithm, spanning inflammatory, metabolism, and EMT programs based on the pathway enriched with their characteristic gene expression signatures. Such shared meta programs are covered by all ccRCC cases in single-cell data. When tracing spatial locations of cancer cells in different meta programs by stRNA-seq, we noticed that cancer cells of the EMT program were uniformly distributed in the margins and perivascular regions of the tumor lesion, while cancer cells of the metabolism program enriched in hypoxia and xenobiotic metabolism-related pathways were mainly located in the center of the tumor lesion, confirming the functional status of various cancer cells. Based on relative proportions of cancer cells of disparate meta programs calculated by CIBERSORTx, the infiltration abundance of cancer cells of the EMT program was associated with worse clinical outcomes of ccRCC patients, while relative fractions of cancer cells of the metabolism program were correlated with favorable prognostic outcomes of ccRCC cases in the TCGA database. This shows that functional classification of cancer cells has a high prognostic value.

scATAC-seq is a state-of-the-art method for revealing epigenetic regulation at the DNA level. Compared to scRNA-seq data analysis, which infers activity changes in transcription factors based on expression profiles of target genes regulated by the transcription factor regulatory network, scATAC-seq can demonstrate the coordinated interactions of transcription factors with arrays of transcription factor binding sites from the DNA level [36, 37]. Regulation of target genes by transcription factors exerts bidirectional effects of transcriptional activation or functional repression. Therefore, it is important to integrate scATAC-seq and scRNA data to infer and mutually validate the regulation of transcription factors of different meta programs. We revealed the transcription factors that regulate the functional characteristics of cancer cells with different meta programs through scATAC and scRNA-seq. We identified the functional characteristic transcription factors that are involved in the regulatory network orchestrated by cancer cells via scATAC-seq and scRNA-seq analyses. The MYC specifically regulated the metabolism programs, while the downstream target genes co-expressed with MYC were enriched in hypoxia-related signaling pathways. The role of MYC in tumor metabolism has been reported. It enhances the expression level of bioenergetic-related genes controlling glucose, glutamine, fatty acid and cholesterol metabolism, thereby activating metabolic reprogramming of cancer cells [38–40]. Our results underlined the pivotal stage of MYC as an upstream oncogene in promoting the metabolic states of ccRCC cells. We noted that transcription factors (EGR1, FOS and JUNB) specifically regulate the EMT meta program, with target genes being enriched in EMT, myogenesis, and angiogenesis signaling pathways. The EGR1 promotes cancer cell EMT by initiating the transcription of E-cadherin transcriptional inhibitors (i.e., SNAIL and SLUG), thereby promoting the invasive and metastatic properties of cancer cells. An identical role of EGR1 has been reported in prostate [41], liver [42], and ovarian cancers [43]. Besides, FOS and JUNB are proto-oncogenes, the proteins of which are key subunits of the AP-1 transcription factor, and are invariably associated with the EMT process [44–46]. In the inflammatory program, we identified a regulatory pattern of the transcription factors (ELF1 and CREM), whose essential roles in immune regulation and inflammatory responses have been reported [47–49]. Their corresponding target genes were found to be enriched in inflammatory responses, complement, interferon gamma responses and the IL2 STAT5 signaling pathway. We tentatively postulated that the effects of transcription factors (ELF1 and CREM) on ccRCC cells are involved in immune regulation, however, the involved molecular mechanisms have yet to be established. The scRNA-seq and scATAC-seq revealed the transcription factor regulatory network of functionally heterogeneous cancer cells during malignant phenotypes and metastasis of ccRCC TME, which is as an integrative avenue facilitating the acquisition of cancer hallmarks and informing cancer precision treatment, e.g., targeted therapy.

Then, we investigated the interactions between functionally heterogeneous cancer cells and cell subpopulations in the TME based on ligand receptor pairs using CellphoneDB. The connecting bonds between ligand molecules and their corresponding receptors on cell surfaces are imperative for exertion of biological functions of certain cell types. We noted that the EMT program presented abundant crosstalks with other cell subpopulations in the TME, while interactions between the metabolism program and other cell subpopulations were markedly mitigated. These findings were corroborated with the spatial localization information provided by stRNA-seq. CellphoneDB indicated that FGFR, as an EMT receptor, may have indispensable effects on functional maintenance of such tumor cells, with which the corresponding ligand cells, especially macrophages and mesenchymal stromal cells, manifested the most abundant crosstalks, elucidating the close association of the two immune cell subtypes with the EMT of cancer cells. These findings are in tandem with those of previous studies [50–52], which reported on the functions of FGFR in mediating EMT and angiogenesis of cancers [31], thereby highlighting the great potential of targeting FGFR, especially the FGFR2 receptor, in reversing the EMT process of ccRCC. The LGALS9-related ligands and receptors are of significance in functionally heterogeneous cancer cells of the inflammatory program, which is involved in cancer cell immune escape. Pharmacological modalities with antagonists or antibodies blocking such interactions may provide a potential and promising strategy for clinical management of ccRCC.

Surgical resection is the preferable treatment option for patients with localized ccRCC. Due to its insidious onset and progressive nature, most cases present terminal with inoperable advanced stages and receive systemic chemotherapies. Benefits from standard chemotherapies are poor, and a majority of the responders develop resistance, resulting in limited survival outcomes. Intra-tumor heterogeneity has a major role in tumor relapse and drug resistance [1, 6, 7, 53]. We analyzed the molecular profiles of key molecules for current targeted therapies in functionally heterogeneous ccRCC cells. We analyzed the molecular profiles of key molecules for mainstream targeted therapies in functionally heterogeneous cancer cells. We found that increased expression of VEGFA in cancer cells of the three types of programs, mediated the benefits of Bevacizumab treatment, a humanized monoclonal antibody against VEGFA, in ccRCC treatment. Particularly, VEGFB, which is a newly identified target sharing similar structures with VEGFA [54], exhibited enhanced expression level in cancer cells. Translational studies have elucidated on the therapeutic potentials of targeting VEGFB. Moreover, CDK4, EGFR, and MAP2K2 exhibited moderately high expression levels in cancer cells, with drugs targeting such therapeutic molecules presenting encouraging results in cancer therapy, but their clinical applications in kidney cancer are rarely reported [55–57]. Apart from molecular targets expressed in cancer cells of the three types of programs, CD52, CDK6, HDAC2, and PIGF were specifically expressed in inflammatory or EMT meta programs, indicating that drugs against these targets may respond in specific cancer cell subpopulations.

This study has various limitations. First, even though we described functionally heterogeneous cancer cells of meta programs that generally present in all three ccRCC cases in the dataset based on the cNMF algorithm, the number and features of meta programs could be somewhat divergent as the sample size was expanded. Second, we provided preliminary insights on potential targets to varying heterogeneous cancer cells, however, the value of such therapeutic targets should be validated in large-scale, multicenter prospective cohorts.

## CONCLUSIONS

Our study revealed the transcriptomic and epigenomic features of functionally heterogeneous cancer cells in ccRCC TME using single-cell multi-omics data, then spatially localized heterogeneous cancer cells with spatial omics, thereby providing preliminary insights into the intra-tumor heterogeneity of ccRCC and its regulatory network. Our findings will inform on development of rational therapeutic strategies.

## MATERIALS AND METHODS

### Data acquisition

Raw data for ccRCC scRNA-seq and scATAC-seq were downloaded from the National Center for Biotechnology Information (NCBI) Sequence Read Archive (SRA; https://www.ncbi.nlm.nih.gov/sra) database with BioProject number PRJNA768891 [23]. After conducting initial quality control on the raw data based on the report of Cell Ranger, we included samples with the following sample IDs in the scRNA-seq dataset: SRR16213611, SRR16213612, and SRR16213614. Additionally, we included samples with the following sample IDs in the scATAC-seq dataset: SRR16213608, SRR16213609, and SRR16213610. Meanwhile, we acquired the scRNA-seq data of kidneys from three human donors as the normal controls from Liao et. al’s study [58]. We also downloaded spatial transcriptomics analysis data performed on ccRCC primary tumors using paraffin-embedded (FFPE) sections from the Gene Expression Omnibus (GEO) database with accession number GSE175540, including four samples with sample IDs GSM5924033, GSM5924035, GSM5924037, and GSM5924040, which were relatively well in histological structure of sections [59]. Bulk RNA-seq data for ccRCC were obtained from the Genomic Data Commons (GDC) portal (https://portal.gdc.cancer.gov/) of The Cancer Genome Atlas (TCGA) database, and after filtering out samples with incomplete follow-up information and those with follow-up information less than one month, 490 cases were included for analysis.

### Processing of scRNA-seq data

The FASTQ raw data files of scRNA-seq were mapped to the reference genome GRCh38-2020-A, and were processed via Cell Ranger (version 6.1.2, 10x Genomics) with default parameters. The Seurat package in R software was used for analyses of scRNA-seq data. The SCTransform, RunPCA and RunUMAP functions were used for dimensionality reduction, clustering, and visualization. Low-quality cells with < 200 or > 5000 covered genes were filtered out, while cells with > 10% mitochondrial RNA contents were also removed. The Harmony (version 1.0) package was used for batch effects removal. Cell-type specific marker genes were identified using the FindAllMarkers function of Seurat package, thus manually annotating each cell type in scRNA-seq.

### scATAC-seq data processing

FASTQ raw data files of scATAC-seq data were processed via Cell Ranger-atac-2.1.0 using default parameters, which were aligned to the human reference genome (GRCh38-2020-A-2.0.0). Then, scATAC-seq was analyzed using the Signac pipeline (version 1.6.0) in R, with the RunT-FIDF, RunSVD, and RunUMAP functions being used for dimensionality reduction and clustering. Further cell filtering was performed as follows: cells with nucleosome signal scores < 4 and transcriptional start site (TSS) enrichment scores > 3. Cell peak region fragments > 1000 or < 20000, and blacklist ratio < 0.05 were retained for subsequent analyses. Batch effects across samples were removed using the Harmony (version 0.1.1) package. Gene activities were quantified via the GeneActivity function in Signac, including 2 kb upstream of the transcriptional start site and gene body. We annotated the cell types in scATAC-seq data according to cell identities in scRNA-seq data using the TransferData function in Seurat package. Chromatin accessibility of the corresponding marker gene was used for cell type annotations. Motif activity scores in each cell were analyzed using the chromVAR (version 1.20.0) package. Transcription factor footprint analysis was conducted using the Footprint function.

### stRNA-seq data processing

Analysis of stRNA data was performed using the Seurat (version 4.2.0) package. Spots with < 300 detected genes or > 30% mitochondrial genes were filtered out. SCTransform, RunPCA and RunUMAP functions were used for normalization and downscale clustering. Cell types in scRNA data were mapped to spatial locations using the TransferData function in Seurat package.

### NMF analysis and module identification

The consensus non-negative matrix factorization (cNMF) algorithm was used to infer gene expression programs from scRNA-Seq data with the cNMF python pipeline (version 1.4) based on count matrix of cancer cells across samples. Each matrix was decomposed into different programs using the cNMF algorithm for 300 iterations. Then, the number of decomposition programs with higher stability and lower error probability was selected by setting the K value, further filtering the inconsistent iterations with the threshold value, thereby choosing the optimal matrix decomposition. After matrix decomposition, cells of programs below a threshold value of 0.03 were excluded. Correlation analyses were performed to identify programs with shared biological functions in different samples. The meta programs were defined via hierarchical clustering with cluster_method=“complete”. Finally, the top 50 genes with the highest loading were defined as functional gene sets for disparate meta programs.

### Single-cell copy number variation analysis

The inferCNV (version 1.10.1) package was used to infer copy number variations of cells based on gene count expression matrix with T cells as the normal reference. This allowed the differentiation of cancer cells from normal cells. The AnnoProbe (version 0.1.6) package was used to generate the reference gene set, containing a gene ordering file from the human GRCh38 assembly with positions of each gene’s chromosomal start and end.

### Pseudotime trajectory analysis

Pseudo-time analysis and reconstruction of the differentiation trajectory of distinct cell lineages were performed using the Monocle (version 2.22.0) package. Count matrix was used as the input matrix while functions within the Monocle package were used for data normalization and preprocessing. Genes with higher dispersion were selected to analyze the differentiation characteristics of specific cell populations. The reduceDimension and orderCells functions were used for dimensionality reduction and cell sorting.

### Identification of specific transcription factors for cancer cells

Analyses of specific transcription factors for cancer cells in different meta programs were performed by integrating scRNA and scATAC data. Regarding the scRNA data, we defined the regulatory networks of transcription factors with target genes in meta programs by pySCENIC. Then, the top 50 transcriptional regulatory networks that are essential for biological functions of each meta program were selected. For the scATAC data, DNA sequence motif analysis of transcription factors involved in regulatory networks was performed using the RunchromVAR function of the chromVAR package, thereby quantifying DNA binding of transcription factors in specific sequences. Finally, transcription factors with program-specific motif activities and footprints were selected to define specific transcriptional regulators of cancer cells in meta programs.

### CIBERSORTx analysis

CIBERSORTx is an emerging machine-learning approach that is designed to assess the abundance of certain cell types in bulk RNA-seq data. This approach has been described as “digital cytometry”. In this study, CIBERSORTx was used to enumerate the relative proportions of cancer cells in meta programs of the ccRCC cohort in the TCGA database. The associations between infiltrating levels of different cancer cell types and survival outcomes of ccRCC cases were determined via Cox analysis and Kaplan-Meier (KM) survival curves, which were performed using survival (version 3.3-1) and survminer (version 0.4.9) packages. The grouping condition of ccRCC samples is based on surv_cutpoint function of survminer R package. The optimal cutoff value is selected, and the sample size with the lowest grouping is set not less than 30% of the total sample size.

### Cell-cell communication analysis

Python-based CellPhoneDB (version 2.0.0) was used to assess crosstalks between cell subpopulations. Normalized data matrices were used for subsequent analyses, with *p* > 0.05 being set as the threshold for excluding ligand-receptor pairs. Radar plots were established to visualize the number of differential cell communications using the ggiraphExtra package (version 0.3.0).

### Functional enrichment analysis

Functional enrichment analysis of the top 50 feature gene sets of meta programs was performed using the enricher function of clusterProfiler package (version 4.2.2), based on the 50 hallmark gene sets downloaded from the Molecular Signatures Database (MSigDB). Adjusted *p* < 0.05 indicated significant differences. Single-sample gene set enrichment analysis (ssGSEA) of the top 50 feature gene sets in the TCGA ccRCC cohort was performed using the GSVA package.

### Immunofluorescence analysis

Tumor tissues were obtained from ccRCC patients who had been subjected to radical resection using a protocol approved by The First Affiliated Hospital of Guangxi Medical University. The paraffin-embedded ccRCC sections were deparaffinized using a dewaxing agent. Then, antigen repair was performed by heating with citric acid solution (Servicebio, G1202), followed by inactivation of endogenous peroxidase using 3% H_2_O_2_. Then, the ccRCC sections were blocked using 3% bovine serum albumin (BSA) (Servicebio, GC305010) for 30 min and thereafter incubated overnight at 4° C with primary antibodies (anti-REGA1 (Abcam, ab47099), anti-IL10RA (Bioss, bs-18131R), or anti-COL1A1 (ZEN BIO, R26615)). The ccRCC sections were washed thrice using PBS, incubated with goat anti-rabbit IgG H&L (HRP) (Servicebio, GB21303) for 50 min and thereafter with CY3-tyramide (Servicebio, G1223) for 10 min. Then, antigen repair was repeatedly performed to remove the first type of primary antibody, followed by overnight incubation at 4° Cin the presence of anti-CA9 (Servicebio, GB112005). The next day, the ccRCC sections were washed using PBS and incubated with anti-rabbit IgG (Alexa Fluor 488 Conjugate) (Servicebio, GB25303) for 50 min. Finally, the sections were stained with DAPI (Servicebio, G1012), and sealed using the anti-fluorescence quencher (Servicebio, G1401).

### Statistical analysis

All data analyses were conducted using the R software (version 4.1.2). Spearman correlations between different cell clusters were calculated using the Cor function of R package stats (version 4.1.2). Clustering of cancer cells was performed using “complete” in R. The Kruskal-Wallis test was performed for differential analysis of infiltration abundance of cancer cells in disparate meta programs. p< 0.05 was the threshold for statistical significance.

### Availability of data and materials

Raw data for ccRCC scRNA-seq as well as scATAC-seq were downloaded from the National Center for Biotechnology Information (NCBI) Sequence Read Archive (SRA; https://www.ncbi.nlm.nih.gov/sra) database with BioProject number PRJNA768891. The transcriptional data of kidneys from three human donors were the normal controls from the Gene Expression Omnibus (GEO) database with accession number GSE131685. Spatial transcriptomics data were performed on ccRCC primary tumors using paraffin-embedded (FFPE) sections from the GEO database with accession number GSE175540. Bulk RNA-seq data for ccRCC were obtained from the Genomic Data Commons (GDC) portal (https://portal.gdc.cancer.gov/) of The Cancer Genome Atlas (TCGA) database.

## Supplementary Material

Supplementary Figures

Supplementary Tables

## References

[1] Hsieh JJ, Purdue MP, Signoretti S, Swanton C, Albiges L, Schmidinger M, Heng DY, Larkin J, Ficarra V. Renal cell carcinoma. Nat Rev Dis Primers. 2017; 3:17009. 10.1038/nrdp.2017.9 28276433 PMC5936048

[2] Sung H, Ferlay J, Siegel RL, Laversanne M, Soerjomataram I, Jemal A, Bray F. Global Cancer Statistics 2020: GLOBOCAN Estimates of Incidence and Mortality Worldwide for 36 Cancers in 185 Countries. CA Cancer J Clin. 2021; 71:209–49. 10.3322/caac.21660 33538338

[3] Rathmell WK, Rumble RB, Van Veldhuizen PJ, Al-Ahmadie H, Emamekhoo H, Hauke RJ, Louie AV, Milowsky MI, Molina AM, Rose TL, Siva S, Zaorsky NG, Zhang T, et al. Management of Metastatic Clear Cell Renal Cell Carcinoma: ASCO Guideline. J Clin Oncol. 2022; 40:2957–95. 10.1200/JCO.22.00868 35728020

[4] Kotecha RR, Motzer RJ, Voss MH. Towards individualized therapy for metastatic renal cell carcinoma. Nat Rev Clin Oncol. 2019; 16:621–33. 10.1038/s41571-019-0209-1 30992569

[5] Barata PC, Rini BI. Treatment of renal cell carcinoma: Current status and future directions. CA Cancer J Clin. 2017; 67:507–24. 10.3322/caac.21411 28961310

[6] Alizadeh AA, Aranda V, Bardelli A, Blanpain C, Bock C, Borowski C, Caldas C, Califano A, Doherty M, Elsner M, Esteller M, Fitzgerald R, Korbel JO, et al. Toward understanding and exploiting tumor heterogeneity. Nat Med. 2015; 21:846–53. 10.1038/nm.3915 26248267 PMC4785013

[7] McGranahan N, Swanton C. Clonal Heterogeneity and Tumor Evolution: Past, Present, and the Future. Cell. 2017; 168:613–28. 10.1016/j.cell.2017.01.018 28187284

[8] Easwaran H, Tsai HC, Baylin SB. Cancer epigenetics: tumor heterogeneity, plasticity of stem-like states, and drug resistance. Mol Cell. 2014; 54:716–27. 10.1016/j.molcel.2014.05.015 24905005 PMC4103691

[9] Beksac AT, Paulucci DJ, Blum KA, Yadav SS, Sfakianos JP, Badani KK. Heterogeneity in renal cell carcinoma. Urol Oncol. 2017; 35:507–15. 10.1016/j.urolonc.2017.05.006 28551412

[10] Ciccarese C, Brunelli M, Montironi R, Fiorentino M, Iacovelli R, Heng D, Tortora G, Massari F. The prospect of precision therapy for renal cell carcinoma. Cancer Treat Rev. 2016; 49:37–44. 10.1016/j.ctrv.2016.07.003 27453294

[11] Dizman N, Philip EJ, Pal SK. Genomic profiling in renal cell carcinoma. Nat Rev Nephrol. 2020; 16:435–51. 10.1038/s41581-020-0301-x 32561872

[12] Cancer Genome Atlas Research Network. Comprehensive molecular characterization of clear cell renal cell carcinoma. Nature. 2013; 499:43–9. 10.1038/nature12222 23792563 PMC3771322

[13] Tang F, Barbacioru C, Wang Y, Nordman E, Lee C, Xu N, Wang X, Bodeau J, Tuch BB, Siddiqui A, Lao K, Surani MA. mRNA-Seq whole-transcriptome analysis of a single cell. Nat Methods. 2009; 6:377–82. 10.1038/nmeth.1315 19349980

[14] Haque A, Engel J, Teichmann SA, Lönnberg T. A practical guide to single-cell RNA-sequencing for biomedical research and clinical applications. Genome Med. 2017; 9:75. 10.1186/s13073-017-0467-4 28821273 PMC5561556

[15] Wang C, He Y, Zheng J, Wang X, Chen S. Dissecting order amidst chaos of programmed cell deaths: construction of a diagnostic model for KIRC using transcriptomic information in blood-derived exosomes and single-cell multi-omics data in tumor microenvironment. Front Immunol. 2023; 14:1130513. 10.3389/fimmu.2023.1130513 37153569 PMC10154557

[16] Neftel C, Laffy J, Filbin MG, Hara T, Shore ME, Rahme GJ, Richman AR, Silverbush D, Shaw ML, Hebert CM, Dewitt J, Gritsch S, Perez EM, et al. An Integrative Model of Cellular States, Plasticity, and Genetics for Glioblastoma. Cell. 2019; 178:835–49.e21. 10.1016/j.cell.2019.06.024 31327527 PMC6703186

[17] Puram SV, Tirosh I, Parikh AS, Patel AP, Yizhak K, Gillespie S, Rodman C, Luo CL, Mroz EA, Emerick KS, Deschler DG, Varvares MA, Mylvaganam R, et al. Single-Cell Transcriptomic Analysis of Primary and Metastatic Tumor Ecosystems in Head and Neck Cancer. Cell. 2017; 171:1611–24.e24. 10.1016/j.cell.2017.10.044 29198524 PMC5878932

[18] Yu Z, Lv Y, Su C, Lu W, Zhang R, Li J, Guo B, Yan H, Liu D, Yang Z, Mi H, Mo L, Guo Y, et al. Integrative Single-Cell Analysis Reveals Transcriptional and Epigenetic Regulatory Features of Clear Cell Renal Cell Carcinoma. Cancer Res. 2023; 83:700–19. 10.1158/0008-5472.CAN-22-2224 36607615 PMC9978887

[19] Barkley D, Moncada R, Pour M, Liberman DA, Dryg I, Werba G, Wang W, Baron M, Rao A, Xia B, França GS, Weil A, Delair DF, et al. Cancer cell states recur across tumor types and form specific interactions with the tumor microenvironment. Nat Genet. 2022; 54:1192–201. 10.1038/s41588-022-01141-9 35931863 PMC9886402

[20] Jia Y, Zhang B, Zhang C, Kwong DL, Chang Z, Li S, Wang Z, Han H, Li J, Zhong Y, Sui X, Fu L, Guan X, Qin Y. Single-Cell Transcriptomic Analysis of Primary and Metastatic Tumor Ecosystems in Esophageal Squamous Cell Carcinoma. Adv Sci (Weinh). 2023; 10:e2204565. 10.1002/advs.202204565 36709495 PMC9982558

[21] Longo SK, Guo MG, Ji AL, Khavari PA. Integrating single-cell and spatial transcriptomics to elucidate intercellular tissue dynamics. Nat Rev Genet. 2021; 22:627–44. 10.1038/s41576-021-00370-8 34145435 PMC9888017

[22] Qi J, Sun H, Zhang Y, Wang Z, Xun Z, Li Z, Ding X, Bao R, Hong L, Jia W, Fang F, Liu H, Chen L, et al. Single-cell and spatial analysis reveal interaction of FAP^+^ fibroblasts and SPP1^+^ macrophages in colorectal cancer. Nat Commun. 2022; 13:1742. 10.1038/s41467-022-29366-6 35365629 PMC8976074

[23] Long Z, Sun C, Tang M, Wang Y, Ma J, Yu J, Wei J, Ma J, Wang B, Xie Q, Wen J. Single-cell multiomics analysis reveals regulatory programs in clear cell renal cell carcinoma. Cell Discov. 2022; 8:68. 10.1038/s41421-022-00415-0 35853872 PMC9296597

[24] Hu J, Chen Z, Bao L, Zhou L, Hou Y, Liu L, Xiong M, Zhang Y, Wang B, Tao Z, Chen K. Single-Cell Transcriptome Analysis Reveals Intratumoral Heterogeneity in ccRCC, which Results in Different Clinical Outcomes. Mol Ther. 2020; 28:1658–72. 10.1016/j.ymthe.2020.04.023 32396851 PMC7335756

[25] Kotliar D, Veres A, Nagy MA, Tabrizi S, Hodis E, Melton DA, Sabeti PC. Identifying gene expression programs of cell-type identity and cellular activity with single-cell RNA-Seq. Elife. 2019; 8:e43803. 10.7554/eLife.43803 31282856 PMC6639075

[26] Qiu B, Ackerman D, Sanchez DJ, Li B, Ochocki JD, Grazioli A, Bobrovnikova-Marjon E, Diehl JA, Keith B, Simon MC. HIF2α-Dependent Lipid Storage Promotes Endoplasmic Reticulum Homeostasis in Clear-Cell Renal Cell Carcinoma. Cancer Discov. 2015; 5:652–67. 10.1158/2159-8290.CD-14-1507 25829424 PMC4456212

[27] Su C, Lv Y, Lu W, Yu Z, Ye Y, Guo B, Liu D, Yan H, Li T, Zhang Q, Cheng J, Mo Z. Single-Cell RNA Sequencing in Multiple Pathologic Types of Renal Cell Carcinoma Revealed Novel Potential Tumor-Specific Markers. Front Oncol. 2021; 11:719564. 10.3389/fonc.2021.719564 34722263 PMC8551404

[28] Acuner-Ozbabacan ES, Engin BH, Guven-Maiorov E, Kuzu G, Muratcioglu S, Baspinar A, Chen Z, Van Waes C, Gursoy A, Keskin O, Nussinov R. The structural network of Interleukin-10 and its implications in inflammation and cancer. BMC Genomics. 2014 (Suppl 4); 15:S2. 10.1186/1471-2164-15-S4-S2 25056661 PMC4083408

[29] Ghosh AK. Factors involved in the regulation of type I collagen gene expression: implication in fibrosis. Exp Biol Med (Maywood). 2002; 227:301–14. 10.1177/153537020222700502 11976400

[30] Zhang F, Yu S, Wu P, Liu L, Wei D, Li S. Discovery and construction of prognostic model for clear cell renal cell carcinoma based on single-cell and bulk transcriptome analysis. Transl Androl Urol. 2021; 10:3540–54. 10.21037/tau-21-581 34733651 PMC8511535

[31] Babina IS, Turner NC. Advances and challenges in targeting FGFR signalling in cancer. Nat Rev Cancer. 2017; 17:318–32. 10.1038/nrc.2017.8 28303906

[32] Golden-Mason L, McMahan RH, Strong M, Reisdorph R, Mahaffey S, Palmer BE, Cheng L, Kulesza C, Hirashima M, Niki T, Rosen HR. Galectin-9 functionally impairs natural killer cells in humans and mice. J Virol. 2013; 87:4835–45. 10.1128/JVI.01085-12 23408620 PMC3624298

[33] Atkins MB, Tannir NM. Current and emerging therapies for first-line treatment of metastatic clear cell renal cell carcinoma. Cancer Treat Rev. 2018; 70:127–137. 10.1016/j.ctrv.2018.07.009 30173085

[34] Cencioni MT, Mattoscio M, Magliozzi R, Bar-Or A, Muraro PA. B cells in multiple sclerosis - from targeted depletion to immune reconstitution therapies. Nat Rev Neurol. 2021; 17:399–414. 10.1038/s41582-021-00498-5 34075251

[35] Chen YP, Yin JH, Li WF, Li HJ, Chen DP, Zhang CJ, Lv JW, Wang YQ, Li XM, Li JY, Zhang PP, Li YQ, He QM, et al. Single-cell transcriptomics reveals regulators underlying immune cell diversity and immune subtypes associated with prognosis in nasopharyngeal carcinoma. Cell Res. 2020; 30:1024–42. 10.1038/s41422-020-0374-x 32686767 PMC7784929

[36] Buenrostro JD, Wu B, Litzenburger UM, Ruff D, Gonzales ML, Snyder MP, Chang HY, Greenleaf WJ. Single-cell chromatin accessibility reveals principles of regulatory variation. Nature. 2015; 523:486–90. 10.1038/nature14590 26083756 PMC4685948

[37] Zhang K, Hocker JD, Miller M, Hou X, Chiou J, Poirion OB, Qiu Y, Li YE, Gaulton KJ, Wang A, Preissl S, Ren B. A single-cell atlas of chromatin accessibility in the human genome. Cell. 2021; 184:5985–6001.e19. 10.1016/j.cell.2021.10.024 34774128 PMC8664161

[38] Liu W, Le A, Hancock C, Lane AN, Dang CV, Fan TW, Phang JM. Reprogramming of proline and glutamine metabolism contributes to the proliferative and metabolic responses regulated by oncogenic transcription factor c-MYC. Proc Natl Acad Sci USA. 2012; 109:8983–88. 10.1073/pnas.1203244109 22615405 PMC3384197

[39] Tambay V, Raymond VA, Bilodeau M. MYC Rules: Leading Glutamine Metabolism toward a Distinct Cancer Cell Phenotype. Cancers (Basel). 2021; 13:4484. 10.3390/cancers13174484 34503295 PMC8431116

[40] Dey P, Kimmelman AC, DePinho RA. Metabolic Codependencies in the Tumor Microenvironment. Cancer Discov. 2021; 11:1067–81. 10.1158/2159-8290.CD-20-1211 33504580 PMC8102306

[41] Kuo PL, Chen YH, Chen TC, Shen KH, Hsu YL. CXCL5/ENA78 increased cell migration and epithelial-to-mesenchymal transition of hormone-independent prostate cancer by early growth response-1/snail signaling pathway. J Cell Physiol. 2011; 226:1224–31. 10.1002/jcp.22445 20945384

[42] Chen HA, Kuo TC, Tseng CF, Ma JT, Yang ST, Yen CJ, Yang CY, Sung SY, Su JL. Angiopoietin-like protein 1 antagonizes MET receptor activity to repress sorafenib resistance and cancer stemness in hepatocellular carcinoma. Hepatology. 2016; 64:1637–51. 10.1002/hep.28773 27530187

[43] Cheng JC, Chang HM, Leung PC. Egr-1 mediates epidermal growth factor-induced downregulation of E-cadherin expression via Slug in human ovarian cancer cells. Oncogene. 2013; 32:1041–49. 10.1038/onc.2012.127 22508482

[44] Debnath P, Huirem RS, Dutta P, Palchaudhuri S. Epithelial-mesenchymal transition and its transcription factors. Biosci Rep. 2022; 42:BSR20211754. 10.1042/BSR20211754 34708244 PMC8703024

[45] Luo YZ, He P, Qiu MX. FOSL1 enhances growth and metastasis of human prostate cancer cells through epithelial mesenchymal transition pathway. Eur Rev Med Pharmacol Sci. 2018; 22:8609–15. 10.26355/eurrev_201812_16624 30575900

[46] Cook DP, Vanderhyden BC. Transcriptional census of epithelial-mesenchymal plasticity in cancer. Sci Adv. 2022; 8:eabi7640. 10.1126/sciadv.abi7640 34985957 PMC8730603

[47] Gallant S, Gilkeson G. ETS transcription factors and regulation of immunity. Arch Immunol Ther Exp (Warsz). 2006; 54:149–63. 10.1007/s00005-006-0017-z 16652219

[48] Seifert LL, Si C, Saha D, Sadic M, de Vries M, Ballentine S, Briley A, Wang G, Valero-Jimenez AM, Mohamed A, Schaefer U, Moulton HM, García-Sastre A, et al. The ETS transcription factor ELF1 regulates a broadly antiviral program distinct from the type I interferon response. PLoS Pathog. 2019; 15:e1007634. 10.1371/journal.ppat.1007634 31682641 PMC6932815

[49] Otero DC, Fares-Frederickson NJ, Xiao M, Baker DP, David M. IFN-β Selectively Inhibits IL-2 Production through CREM-Mediated Chromatin Remodeling. J Immunol. 2015; 194:5120–28. 10.4049/jimmunol.1403181 25888642 PMC4433809

[50] May AM, Batoon L, McCauley LK, Keller ET. The Role of Tumor Epithelial-Mesenchymal Transition and Macrophage Crosstalk in Cancer Progression. Curr Osteoporos Rep. 2023; 21:117–27. 10.1007/s11914-023-00780-z 36848026 PMC10106416

[51] Li X, Chen L, Peng X, Zhan X. Progress of tumor-associated macrophages in the epithelial-mesenchymal transition of tumor. Front Oncol. 2022; 12:911410. 10.3389/fonc.2022.911410 35965509 PMC9366252

[52] Zhang J, Hu Z, Horta CA, Yang J. Regulation of epithelial-mesenchymal transition by tumor microenvironmental signals and its implication in cancer therapeutics. Semin Cancer Biol. 2023; 88:46–66. 10.1016/j.semcancer.2022.12.002 36521737 PMC10237282

[53] Graham J, Dudani S, Heng DYC. Prognostication in Kidney Cancer: Recent Advances and Future Directions. J Clin Oncol. 2018. [Epub ahead of print]. 10.1200/JCO.2018.79.0147 30372388

[54] Holmes DI, Zachary I. The vascular endothelial growth factor (VEGF) family: angiogenic factors in health and disease. Genome Biol. 2005; 6:209. 10.1186/gb-2005-6-2-209 15693956 PMC551528

[55] Fassl A, Geng Y, Sicinski P. CDK4 and CDK6 kinases: From basic science to cancer therapy. Science. 2022; 375:eabc1495. 10.1126/science.abc1495 35025636 PMC9048628

[56] Martinelli E, Morgillo F, Troiani T, Ciardiello F. Cancer resistance to therapies against the EGFR-RAS-RAF pathway: The role of MEK. Cancer Treat Rev. 2017; 53:61–9. 10.1016/j.ctrv.2016.12.001 28073102

[57] Caunt CJ, Sale MJ, Smith PD, Cook SJ. MEK1 and MEK2 inhibitors and cancer therapy: the long and winding road. Nat Rev Cancer. 2015; 15:577–92. 10.1038/nrc4000 26399658

[58] Liao J, Yu Z, Chen Y, Bao M, Zou C, Zhang H, Liu D, Li T, Zhang Q, Li J, Cheng J, Mo Z. Single-cell RNA sequencing of human kidney. Sci Data. 2020; 7:4. 10.1038/s41597-019-0351-8 31896769 PMC6940381

[59] Meylan M, Petitprez F, Becht E, Bougoüin A, Pupier G, Calvez A, Giglioli I, Verkarre V, Lacroix G, Verneau J, Sun CM, Laurent-Puig P, Vano YA, et al. Tertiary lymphoid structures generate and propagate anti-tumor antibody-producing plasma cells in renal cell cancer. Immunity. 2022; 55:527–41.e5. 10.1016/j.immuni.2022.02.001 35231421

