# Supplementary material for: Unfolding the mysteries of heterogeneity from a high-resolution perspective: integration analysis of single-cell multi-omics and spatial omics revealed functionally heterogeneous cancer cells in ccRCC
Source: Aging (Albany NY). 2024 Jun 26;16(13):10943–71. doi: 10.18632/aging.205974 (PMC11272124; doi:10.18632/aging.205974)
Supplement: Supplementary Figures [file aging-16-205974-s001.pdf]

SUPPLEMENTARY FIGURES

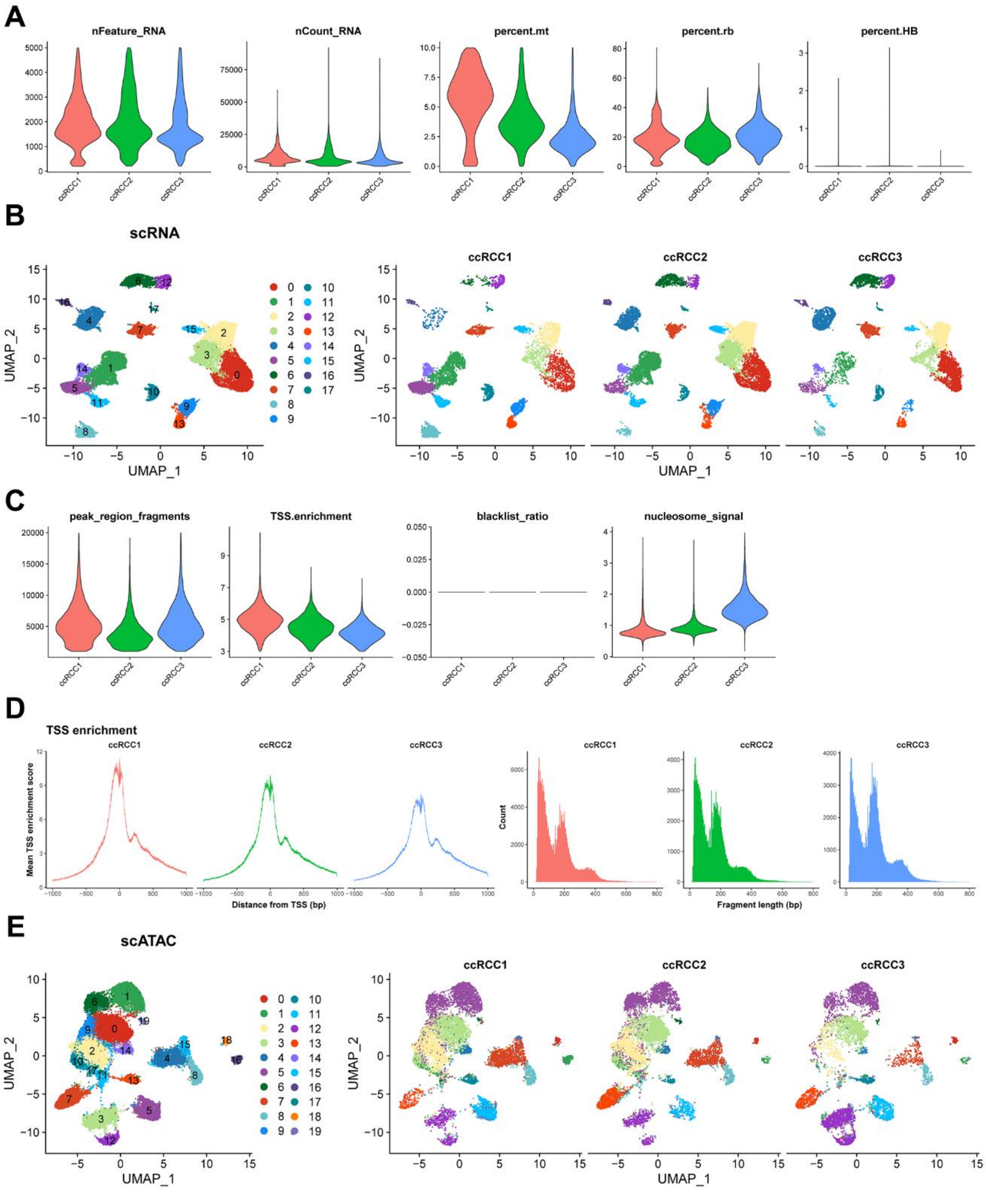

Supplementary Figure 1. Quality control and sample integration of scRNA-seq and scATAC-seq data. (**A**) Violin plots showing quality control indices of scRNA-seq data, including the number of genes detected per cell, count of reads per cell, percentage of mitochondrial genes detected per cell, percentage of ribosomal genes detected per cell, and percentage of hemoglobin genes detected per cell. (**B**) Sample integration of scRNA-seq data. (**C**) Violin plots showing quality control indices of scATAC-seq data, including nucleosome signal scores per cell, TSS enrichment scores per cell, blacklist ratio, and fractions of reads in peaks. (**D**) TSS enrichment score (left) and fragment length (right) of each sample in scATAC-seq data. (**E**) Sample integration of scATAC-seq data.

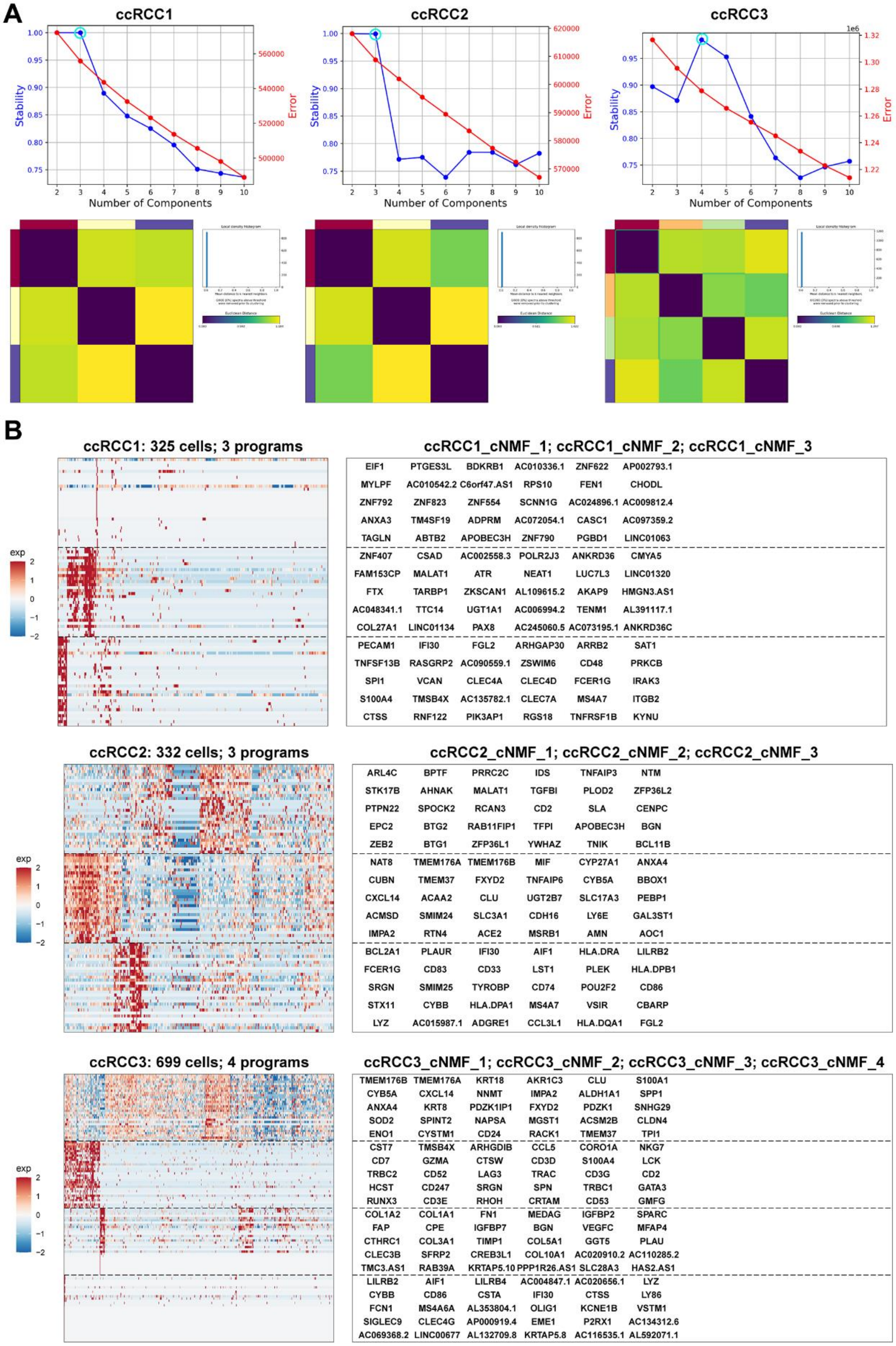

Supplementary Figure 2. Identification of functionally heterogeneous cancer cells in ccRCC based on cNMF algorithm. (**A**) Stability and error curve for inferring gene expression program numbers. The blue circle indicates the optimal number of gene expression program per sample, while the heatmap shows Euclidean distance of programs across replicates. (**B**) Heatmap showing characteristic gene expression of programs in each sample. Right box shows the characteristic gene of different programs.

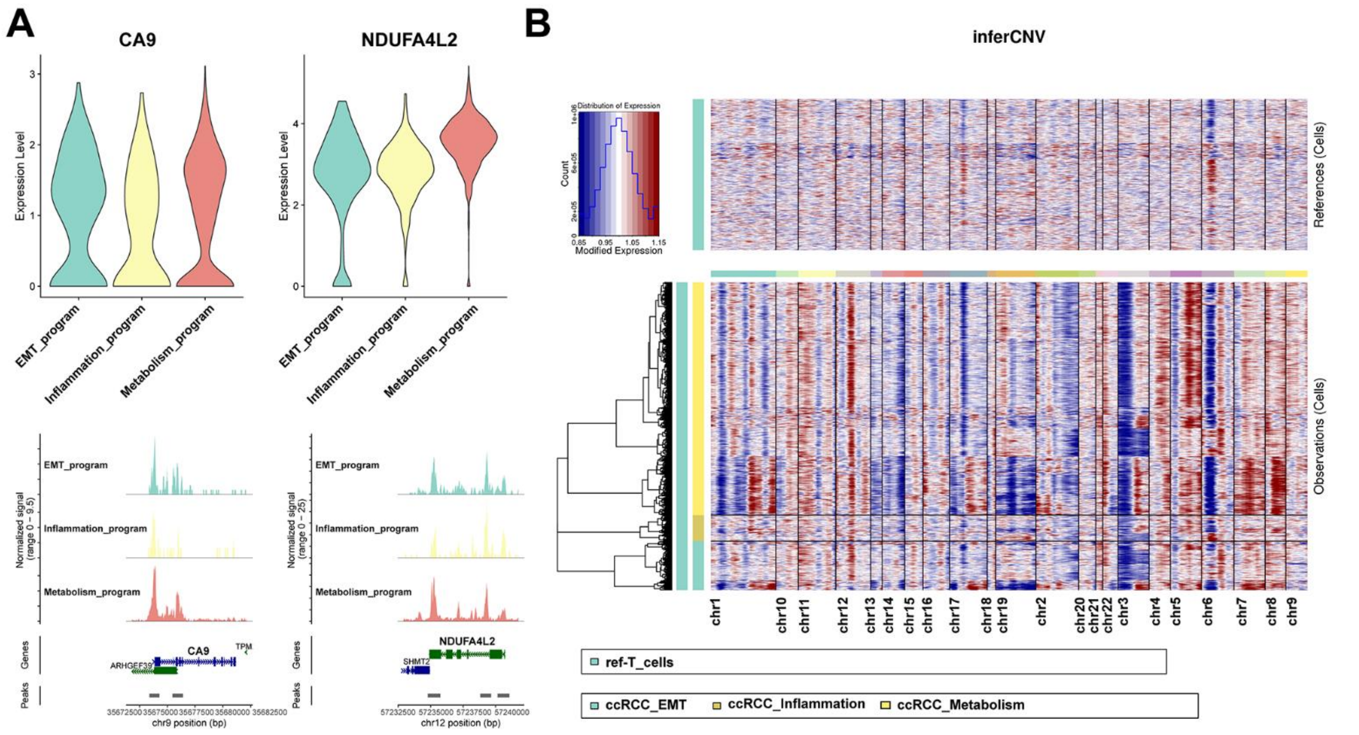

Supplementary Figure 3. Molecular characteristics of functionally heterogeneous cancer cells of different meta programs in ccRCC. (**A**) Transcriptional expression levels and chromatin accessibility of CA9 and NDUFA4L2 in different meta programs of cancer cells. (**B**) Heatmap showing large-scale copy number variations for cancer cells. Red indicates amplification while blue indicates deletion. T cells were used as reference cells to infer copy number variations of cancer cells.

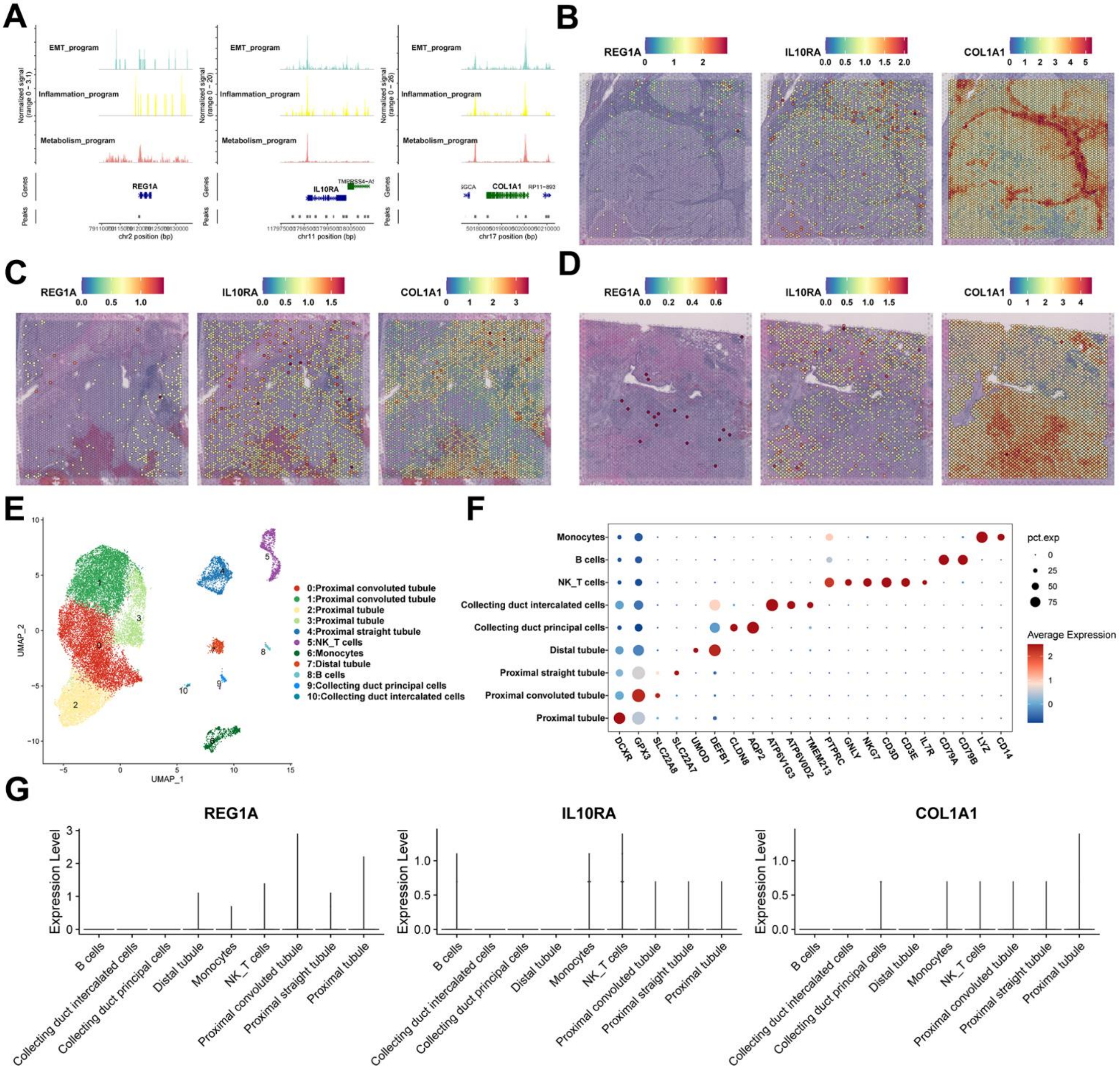

Supplementary Figure 4. Expression profiles of signature genes in heterogeneous cancer cells of three meta programs and in normal kidney cells. (**A**) Chromatin accessibility of REGA1, IL10RA and COL1A1 in three meta programs. (**B**–**D**) Spatial expression patterns for REGA1, IL10RA and COL1A1 in stRNA-seq data. (**E**) UMAP embedding of cells from normal kidneys in scRNA-seq data. (**F**) Dot plot for expression patterns of marker genes for different cell types of normal kidneys in scRNA-seq data. (**G**) Expression levels of REGA1, IL10RA and COL1A1 for different cell types from normal kidneys in scRNA-seq data.

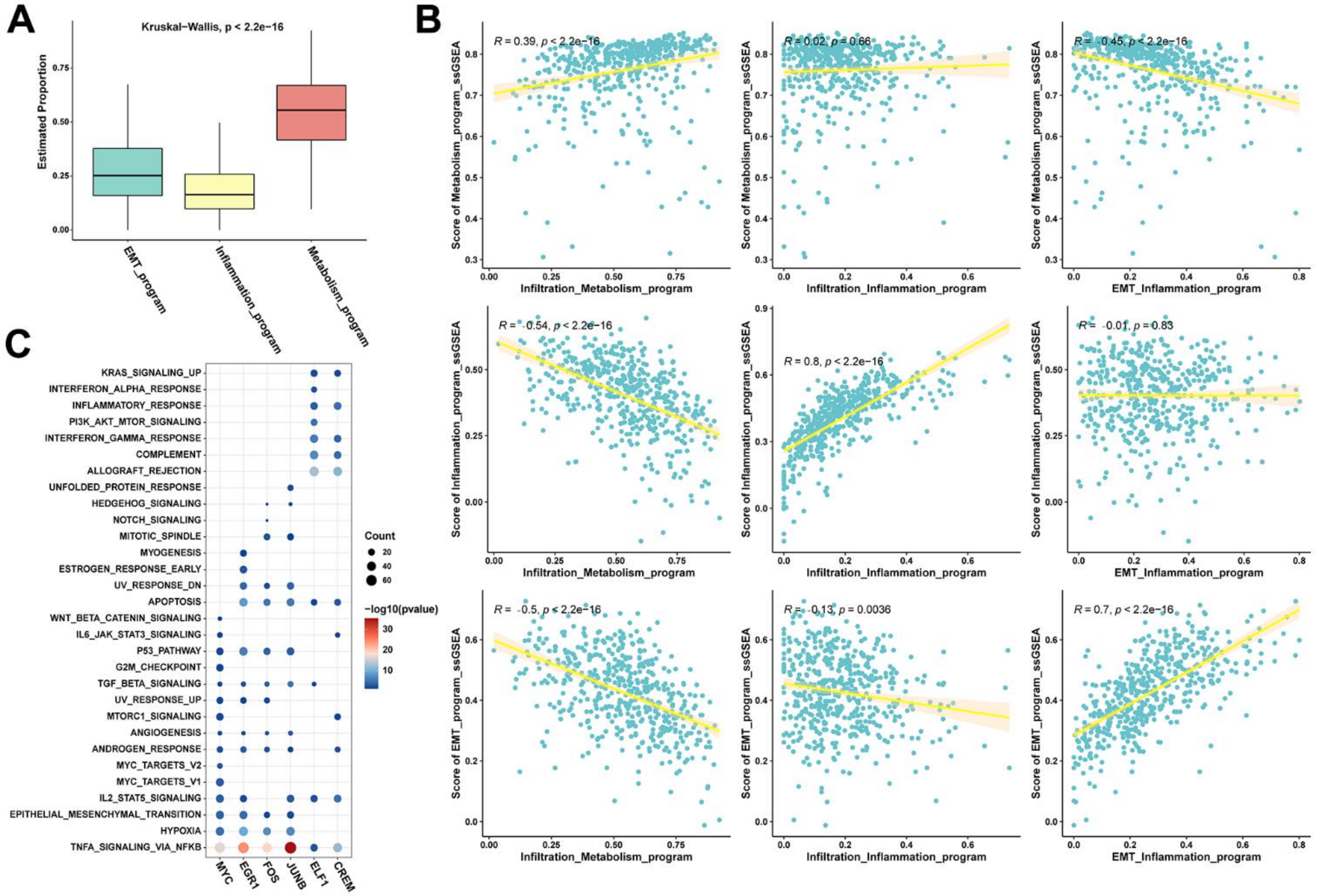

Supplementary Figure 5. Characteristics of heterogeneous cancer cells and associated transcription factor regulatory networks. (**A**) Estimated proportions and differential analysis of relative abundance of functionally heterogeneous cancer cells calculated by CIBERSORTx. (**B**) Correlations between infiltration level of heterogeneous cancer cells of disparate meta programs calculated by CIBERSORTx and ssGSEA scores calculated by characteristic genes of meta programs. (**C**) Functional enrichment analysis results of downstream target genes of specific transcription factors in disparate meta programs.

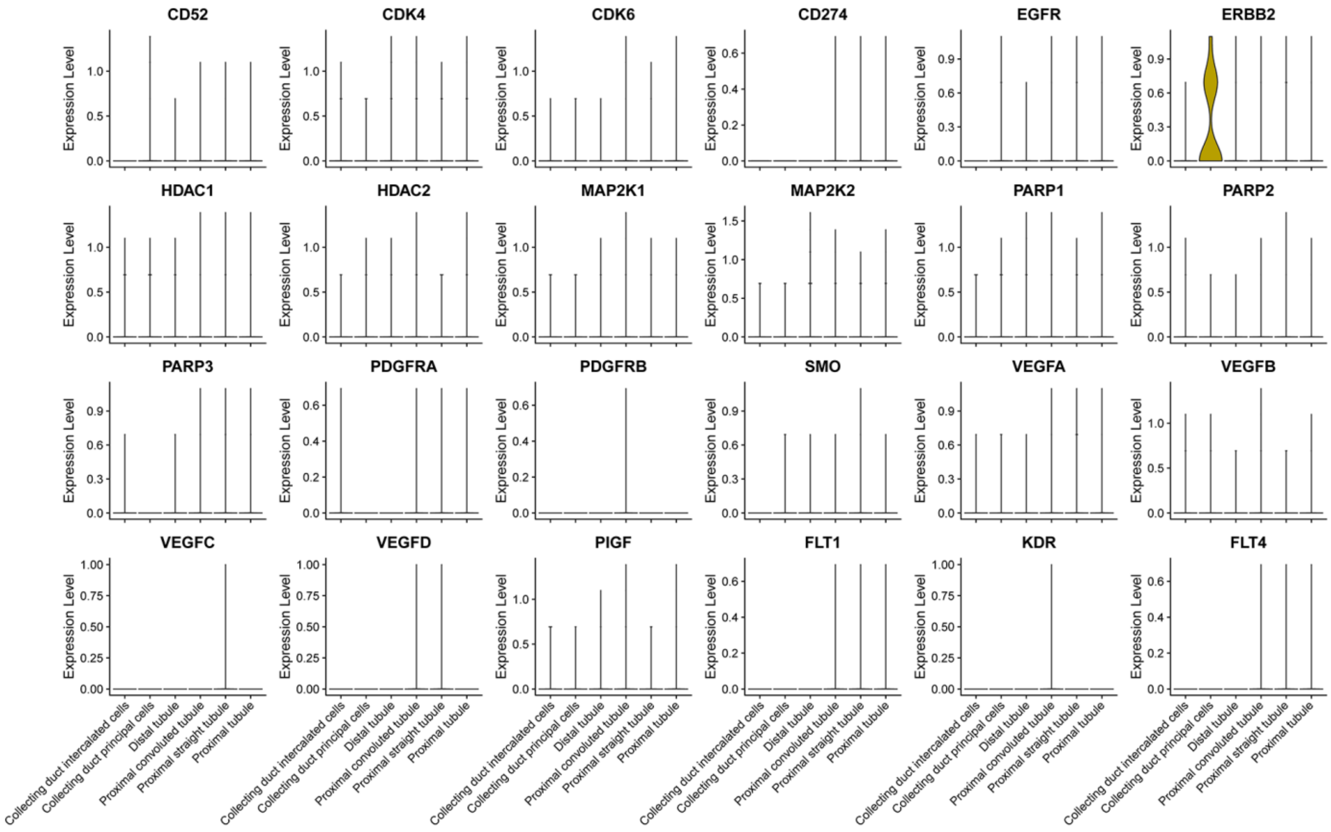

Supplementary Figure 6. Expression level of therapeutic target genes in normal kidneys based on scRNA-seq data.
