# Supplementary material for: Unfolding the mysteries of heterogeneity from a high-resolution perspective: integration analysis of single-cell multi-omics and spatial omics revealed functionally heterogeneous cancer cells in ccRCC
Source: Aging (Albany NY). 2024 Jun 26;16(13):10943–71. doi: 10.18632/aging.205974 (PMC11272124; doi:10.18632/aging.205974)
Supplement: Supplementary Tables [file aging-16-205974-s002.pdf]

SUPPLEMENTARY TABLES

Supplementary Table 1. Data source and quality control in this study.

Data type	Tissue type	Database	Data ID	Sample ID	Sample number	Quality control	Cell number
scRNA	ccRCC	SRA	PRJNA768891	SRR16213611, SRR16213612, SRR16213614	3	minGene>200, maxGene<5000, pctMT<10	27644
scRNA	Normal Kidney	GEO	GSE131685	GSM4145204, GSM4145205, GSM4145206	3	minGene>200, maxGene<2500, pctMT<30	22960
scATAC	ccRCC	SRA	PRJNA768891	SRR16213608, SRR16213609, SRR16213610	3	peak_region_fragments > 1000, peak_region_fragments < 20000, blacklist_ratio < 0.05, nucleosome_signal < 4, TSS.enrichment > 3	26301
stRNA	ccRCC	GEO	GSE175540	GSM5924033, GSM5924035, GSM5924037, GSM5924040	4	minGene>300, pctMT<30	17871
bulk-RNA	ccRCC	GDC-TCGA	-	-	490	-	-

Supplementary Table 2. Functional enrichment analysis of HALMARK term for characteristic gene of meta programs.

ID	GeneRatio	geneID	Count	Cluster
GLYCOLYSIS	9/36	CYB5A/ENO1/MIF/TPI1/TXN/CLDN3/GAL3ST1/PGK1/PKM	9	Metabolism_program
XENOBIOTIC_METABOLISM	6/36	TMEM176B/CYB5A/CYP27A1/SPINT2/PGRMC1/ECH1	6	Metabolism_program
REACTIVE_OXYGEN_SPECIES_PATHWAY	3/36	TXN/MGST1/PRDX2	3	Metabolism_program
HYPOXIA	5/36	ENO1/MIF/TPI1/GAPDH/PGK1	5	Metabolism_program
FATTY_ACID_METABOLISM	4/36	MIF/ACAA2/ALDH1A1/ECH1	4	Metabolism_program
INFLAMMATORY_RESPONSE	25/9	CYBB/PLAUR/IL1B/IL10RA/CD48/GPR183/PDE4B/TNFRSF1B/ADGRE1	9	Inflammation_program
KRAS_SIGNALING_UP	25/9	FCER1G/CTSS/PLAUR/LAPTM5/CD37/IL1B/IL10RA/CLEC4A/TNFRSF1B	9	Inflammation_program
IL2_STAT5_SIGNALING	6/25	FGL2/CD86/IL10RA/CD83/CD48/TNFRSF1B	6	Inflammation_program
ALLOGRAFT_REJECTION	6/25	SRGN/CTSS/CD86/IL1B/LY86/SPI1	6	Inflammation_program
TNFA_SIGNALING_VIA_NFKB	6/25	PLAUR/IL1B/CD83/GPR183/PDE4B/PLEK	6	Inflammation_program
COMPLEMENT	5/25	FCER1G/CTSS/PLAUR/FCN1/PLEK	5	Inflammation_program
INTERFERON_GAMMA_RESPONSE	5/25	IFI30/FGL2/CD86/IL10RA/PDE4B	5	Inflammation_program
EPITHELIAL_MESENCHYMAL_TRANSITION	18/23	COL1A2/COL1A1/FN1/FAP/BGN/SPARC/IGFBP2/CTHRC1/COL5A1/VEGFC/COL3A1/COL6A3/COL6A2/MMP2/HTRA1/PTHLH/TIMP1/SPOCK1	18	EMT_program
COAGULATION	7/23	FN1/SPARC/PLAU/MMP2/C1R/HTRA1/TIMP1	7	EMT_program
MYOGENESIS	6/23	COL1A1/SPARC/IGFBP7/COL3A1/COL6A3/COL6A2	6	EMT_program
UV_RESPONSE_DN	4/23	COL1A2/COL1A1/IGFBP5/COL3A1	4	EMT_program
ANGIOGENESIS	2/23	COL3A1/TIMP1	2	EMT_program

Supplementary Table 3. Top 50 specific characteristic gene of meta programs.

Inflammatory meta programs	EMT meta programs	Metabolism meta programs
LILRB2	COL1A2	TMEM176B
IFI30	COL1A1	TMEM176A
FCER1G	FN1	NAT8
AIF1	FAP	ANXA4
TMSB4X	MFAP4	CYB5A
SRGN	BGN	ENO1
S100A4	AC110285.2	MIF
TYROBP	SPARC	CXCL14
CYBB	CPE	IMPA2
HLA.DRA	GGT5	TPI1
LYZ	IGFBP7	FXYD2
CTSS	IGFBP2	PEBP1
PLAUR	SFRP2	CLU
FGL2	PPP1R26.AS1	TMEM37
CSTA	MEDAG	ACAA2
CD86	IGFBP5	CYP27A1
LAPTM5	CTHRC1	GAPDH
CORO1A	COL5A1	HINT1
COTL1	PLTP	UGT2B7
STX11	PLAU	LY6E
MS4A6A	VEGFC	ALDH1A1
HLA.DPA1	COL3A1	TXN
ARHGDIB	AL359541.1	CLDN3
HLA.DPB1	GJB2	RACK1
GMFG	OLFM2	SPINT2
CD37	SLC28A3	BBOX1
CLEC7A	CLEC3B	BNIP3
IL1B	HAS2.AS1	TNFAIP6
LY86	COL10A1	GAL3ST1
IL10RA	COL6A3	SLC17A3
SPI1	CREB3L1	NDUFA4L2
LILRB4	COL6A2	PGK1
CLEC4A	ARL4C	PDZK1IP1
CD83	AC124067.2	LINP1
CD48	TMC3.AS1	PKM
CD53	AC020910.2	SMIM24
LST1	MMP2	PGRMC1
FCN1	POPDC3	AZGP1
GPR183	C1R	CUBN
PDE4B	HTRA1	MGST1
PLEK	GAP43	ITM2B
HLA.DRB1	PTHLH	SLC3A1
TNFRSF1B	P4HA3	PRDX2
C1orf162	KRTAP5.10	KRT18
VSTM1	TIMP1	RTN4
BTK	SPOCK1	SNHG29
LILRA5	LGI2	MPC2
ABI3	RAB39A	COX6A1
PILRA	RRS1.AS1	ECH1
ADGRE1	DPT	ACMSD
